# Impact of the longitudinal evolution of impaired fasting glucose on cardio-kidney-metabolic multimorbidity

**DOI:** 10.3389/fendo.2026.1812122

**Published:** 2026-06-16

**Authors:** Guanlin Chen, Hong Zheng, Yuxian Wang, Man Gui, Zhen He, Zekai Chen, Shouling Wu, Youren Chen

**Affiliations:** 1 Department of Cardiology, Second Affiliated Hospital of Shantou University Medical College, Shantou, Guangdong, China; 2 School of Nursing and Rehabilitation, Shandong University, Jinan, China; 3 Department of Cardiology, Kailuan General Hospital, Tangshan, China

**Keywords:** cardio-kidney-metabolic multimorbidity, glycemic evolution, glycemic history, impaired fasting glucose, longitudinal cohort, prediabetes

## Abstract

**Aims:**

To characterize longitudinal impaired fasting glucose (IFG) evolution patterns and evaluate their associations with the risk of incident cardio-kidney-metabolic (CKM) multimorbidity.

**Methods:**

In this prospective cohort study, 43,073 adults from the Kailuan Study who underwent examinations in 2006 and 2010 and were free of cardiovascular disease, type 2 diabetes, and chronic kidney disease at baseline were included. IFG status was classified as sustained normal, progression, recovery, or persistent IFG. Participants were followed through 2021. The primary outcome was multimorbidity, defined as ≥2 of the following: cardiovascular disease, type 2 diabetes, or chronic kidney disease. Multivariable Cox models estimated adjusted hazard ratios (HRs).

**Results:**

Over a median follow-up of 11.0 years, 1,795 participants developed CKM multimorbidity. Compared with sustained normal fasting glucose, adjusted HRs (95% CIs) were 1.93 (1.72–2.17) for IFG progression, 1.69 (1.44–1.98) for IFG recovery, and 3.10 (2.74–3.52) for persistent IFG. Using IFG recovery as the reference, risks remained lower for sustained fasting glucose health (HR 0.59; 95% CI 0.51–0.69), whereas persistent IFG remained associated with a higher risk (HR 1.84; 95% CI 1.55–2.18). The association for IFG progression was attenuated and no longer statistically significant (HR 1.14; 95% CI 0.97–1.35).

**Conclusions:**

Individuals who recover from IFG have a lower risk than those with persistent or progressive IFG, yet their risk remains higher than that of individuals with sustained fasting glucose health. These findings highlight that preventing the onset of IFG should be the optimal strategy, followed by timely intervention after its occurrence to mitigate long-term multisystem damage.

## Research in context

**What is already known about this subject?**

Impaired fasting glucose (IFG) is associated with increased risks of type 2 diabetes, cardiovascular disease, and chronic kidney disease when assessed at a single time point.

Prediabetes contributes to adverse cardiometabolic outcomes, but most studies focus on single-organ endpoints rather than multimorbidity.

Evidence on how longitudinal changes in IFG influence long-term, multisystem outcomes remains limited.

**What is the key question?**

How do different longitudinal evolution patterns of impaired fasting glucose affect the risk of developing cardio-kidney-metabolic multimorbidity?

**What are the new findings?**

All IFG evolution patterns (progression, recovery, and persistence) were associated with a higher risk of cardio-kidney-metabolic multimorbidity compared with sustained normal fasting glucose.

Persistent IFG conferred the highest risk, while IFG recovery reduced risk compared with progression or persistence but did not normalize it.

Any history of IFG was associated with long-term excess risk, highlighting cumulative metabolic exposure.

**How might this impact on clinical practice in the foreseeable future?**

These findings support early prevention of IFG and sustained maintenance of normal fasting glucose, along with long-term, integrated cardio-kidney-metabolic risk monitoring even after apparent glycemic recovery.

## Introduction

Driven by shifts in lifestyle behaviors and the aging of populations worldwide, the global burden of prediabetes is substantial and continues to increase. Prediabetes is an intermediate metabolic state between normal glucose homeostasis and overt diabetes, characterized primarily by impaired fasting glucose (IFG) and/or impaired glucose tolerance (IGT). IFG is defined as elevated fasting plasma glucose levels that fall below the diagnostic threshold for diabetes, typically ranging from 5.6 to 6.9 mmol/L according to the American Diabetes Association (ADA) criteria (1). IFG reflects underlying hepatic insulin resistance and early β-cell dysfunction, and individuals with IFG have a markedly increased risk of progression to type 2 diabetes and cardiovascular disease (CVD) (2). As of 2021, the global prevalence of IFG was approximately 5.8% (298 million individuals) and is projected to increase to 6.5% (414 million) by 2045 (3). A nationally representative survey conducted in China in 2018 reported a prediabetes prevalence of 38.1%, while the proportion of individuals achieving adequate management or glycemic control remained low (4). Collectively, these findings indicate that the global burden associated with abnormal glucose metabolism continues to increase.

Meanwhile, the multimorbidity of chronic diseases has become increasingly prevalent. Cardio-kidney-metabolic multimorbidity generally refers to the coexistence of at least two chronic conditions within an individual, specifically cardiovascular disease, chronic kidney disease, or metabolic disorders (5). A large Chinese cohort study published in 2022 reported that approximately 6.0% of participants had multiple cardiometabolic conditions (6). Similarly, between 2015 and 2020, an estimated 20.1 million adults in the United States had two or more cardiac, renal, or metabolic conditions (7). Numerous studies have demonstrated a strong association between multimorbidity and a wide range of adverse health outcomes. For example, each additional chronic condition (e.g., hypertension, diabetes, or heart disease) is associated with a 15% higher risk of dementia (8). Likewise, the coexistence of cardiovascular disease and anemia is strongly linked to a markedly elevated prevalence of frailty (39%) (9).

With respect to the relationship between IFG and components of cardio-kidney-metabolic multimorbidity, a meta-analysis reported that both IFG defined by the American Diabetes Association and IFG defined by the World Health Organization were associated with an increased risk of composite cardiovascular disease (2). Furthermore, even fasting glucose levels within the normoglycemic range exhibit a J-shaped association with the risk of type 2 diabetes (10). In addition, prediabetes has been positively associated with an increased risk of chronic kidney disease, although the strength of evidence and the causal nature of this association require further clarification (11–13). However, most existing studies have focused on single time-point measurements of IFG, hindering comprehensive characterization of its long-term pathological trajectories (14, 15). Moreover, previous research has primarily focused on single-organ outcomes (16, 17), and evidence regarding cardiometabolic–renal multimorbidity as a composite endpoint, as well as the coordinated evolution of its components, remains limited (18). This study aims to identify longitudinal evolution patterns of IFG and to evaluate their associations with the risk of incident cardio-kidney-metabolic multimorbidity and its individual components. In doing so, it seeks to provide evidence-based insights and potential targets for early stratification and intervention within the cardiovascular-kidney-metabolic axis, while addressing evidence gaps inherent in single time-point exposures and single-organ endpoints.

## Methods

### Study design and population

Study participants were drawn from the Kailuan Cohort Study, an ongoing prospective cohort initiated in 2006. Every two years, participants undergo standardized questionnaire surveys, clinical examinations, and laboratory tests at 11 hospitals affiliated with the Kailuan Group. The detailed study design and methods have been described previously (19). The study was conducted in accordance with the Declaration of Helsinki and was approved by the Ethics Committee of Kailuan General Hospital. The present analysis included participants who underwent health examinations in both 2006 and 2010. In total, 68,150 participants completed data collection at both time points. Participants were excluded if they lacked fasting blood glucose data in either 2006 or 2010 (n = 935) or had been diagnosed with cardiovascular disease (n = 1,447), diabetes (n = 11,387), or chronic kidney disease (n = 11,308) in 2010 or earlier. Ultimately, 43,073 participants were included in the final analytic cohort (**Supplementary Figure 1**).

### Assessment of IFG evolution groups

IFG was defined according to the diagnostic criteria of the ADA. Non-pregnant individuals with fasting glucose levels of 5.6–6.9 mmol/L were classified as having IFG (1). Participants were categorized into four IFG evolution groups based on their glycemic status in 2006 and 2010:

1. Sustained normal fasting glucose group: Not diagnosed with IFG at either the 2006 or 2010 examinations.
2. IFG progression group: Not diagnosed with IFG in 2006 but newly diagnosed with IFG in 2010.
3. IFG recovery group: Diagnosed with IFG in 2006 but not diagnosed with IFG in 2010.
4. Persistent IFG group: Diagnosed with IFG at both the 2006 and 2010 examinations.

### Ascertainment of outcome

The primary outcome was cardio-kidney-metabolic multimorbidity, defined as the coexistence of two or more of the following three conditions in an individual: cardiovascular disease (CVD), type 2 diabetes (T2D), or chronic kidney disease (CKD). For participants who developed at least two of the predefined conditions during follow-up, the time of incident multimorbidity was operationally defined as the date of diagnosis of the second condition among CVD, T2D, and CKD. Secondary outcomes included specific combinations of comorbidities: CVD–T2D, CKD–T2D, and CVD–CKD. In addition, associations with each individual disease were examined as supplementary analyses. CVD included myocardial infarction, heart failure, atrial fibrillation, and stroke (ischemic stroke, hemorrhagic stroke, or subarachnoid hemorrhage). Cardiovascular events were identified using International Classification of Diseases, 10th Revision (ICD-10), codes: I21 for myocardial infarction, I60–I61 and I63 for stroke, I48 for atrial fibrillation, and I50 for heart failure (20). Type 2 diabetes and CKD were assessed at each health examination. Type 2 diabetes was defined as fasting blood glucose (FBG) ≥7.0 mmol/L, self-reported physician diagnosis, or current use of antidiabetic medication (1). CKD was defined for epidemiological outcome ascertainment as an estimated glomerular filtration rate (eGFR) <60 mL/min/1.73 m² and/or urine dipstick proteinuria ≥1+ (21–23). eGFR was calculated using the Chronic Kidney Disease Epidemiology Collaboration equation (24).

### Covariate assessment

Participants fasted for at least 8 hours before venous blood samples were collected from the antecubital vein during the morning physical examination. After centrifugation, serum was isolated for the measurement of serum creatinine (Scr), fasting blood glucose (FBG), triglycerides (TG), high-density lipoprotein cholesterol (HDL-C), low-density lipoprotein cholesterol (LDL-C), total cholesterol (TC), and high-sensitivity C-reactive protein (hs-CRP) using an automated biochemical analyzer (Hitachi 7600) at the central laboratory of Kailuan Hospital. FBG was measured using the hexokinase/glucose-6-phosphate dehydrogenase method (coefficient of variation <2.0%), whereas TG and HDL-C were assessed using enzymatic colorimetric assays. Random midstream morning urine samples were also obtained and analyzed with an N-600 urine analyzer (Dirui Medical Technology Co., Ltd., Changchun, China), with semi-quantitative proteinuria categorized as negative (<15 mg/dL), trace (15–29 mg/dL), 1+ (30–300 mg/dL), 2+ (300–1000 mg/dL), or 3+ (>1000 mg/dL). Height, weight, and waist circumference were measured using standardized procedures, and blood pressure was assessed in the right arm with a mercury sphygmomanometer between 7:00 and 9:00 a.m. after at least 5 minutes of rest; three readings obtained at 1–2-minute intervals were averaged for analysis. Beginning in 2014, blood pressure was measured using an HEM-8102A electronic sphygmomanometer (Omron Dalian Co., Ltd., China), with good concordance previously demonstrated between mercury and electronic devices. Additional epidemiological information, including age, sex, educational level, smoking and alcohol consumption, physical activity, and medication use (antihypertensive, lipid-lowering, and hypoglycemic agents), was collected through standardized questionnaires administered by trained personnel.

### Statistical analyses

Baseline characteristics were compared across groups according to the longitudinal evolution of IFG status. Normally distributed continuous variables were presented as mean ± standard deviation (SD) and compared using one-way analysis of variance (ANOVA). Non-normally distributed continuous variables were summarized as median with interquartile range (Q1–Q3) and compared using the Kruskal–Wallis test. The normality of continuous variables was assessed using the Kolmogorov–Smirnov test. Categorical variables were reported as counts and percentages, and differences between groups were assessed using the chi-square test.

The follow-up period for each participant was calculated from the date of the 2010 health examination until the occurrence of cardio-kidney-metabolic multimorbidity, death, or December 31, 2021, whichever occurred first. The proportional hazards assumption was evaluated using Schoenfeld residuals (**Supplementary Table 1**). Missing covariate data (**Supplementary Table 2**) in the Cox models were imputed using multiple imputation by chained equations. Restricted cubic splines were incorporated into Cox proportional hazards regression models to evaluate the potential nonlinear association between fasting blood glucose and the risk of CKM multimorbidity (**Supplementary Figure 2**). Unadjusted cumulative incidence curves were generated using R version 4.5.2. Using the sustained normal fasting glucose group as the reference, Cox proportional hazards models were used to estimate hazard ratios (HRs) and 95% confidence intervals (CIs) for the association between IFG evolution and the risk of cardio-kidney-metabolic multimorbidity. Model 1 was an unadjusted crude model. Model 2 was further adjusted for age, sex, education level, drink status, smoking status and physical activity. Model 3 was further adjusted for body mass index, high-density lipoprotein cholesterol, low-density lipoprotein cholesterol and high-sensitivity C-reactive protein. Model 4 was further adjusted for antihypertensive drugs and lipid-lowering drugs. Similarly, using the IFG recovery group as the reference, we estimated HRs and 95% CIs using the IFG recovery group as the reference to compare risk patterns across IFG evolution groups. In addition, to assess potential time-varying associations, we performed follow-up time-stratified analyses by estimating HRs separately within predefined follow-up intervals. To better quantify the clinical impact, we additionally estimated multivariable-adjusted absolute risk (AR), absolute risk difference (ARD), and the number needed to harm (NNH) using the R package *survival*. Adjusted ARs were estimated using predictive margins derived from multivariable Cox proportional hazards models. A 10-year time horizon was prespecified to provide a clinically interpretable estimate of long-term AR. For each IFG evolution group, exposure status was set to that group for all participants while retaining their observed covariate values, and the predicted 10-year cumulative incidence was then averaged across the study population to obtain the standardized AR. ARDs were calculated by subtracting the standardized AR in the sustained normal fasting glucose group from that in each IFG evolution group. The NNH was calculated as the inverse of the ARD and interpreted as the number of individuals who would need to be exposed to a given IFG evolution pattern, rather than sustained normal fasting glucose, for one additional case of cardio-kidney-metabolic multimorbidity to occur over 10 years. Ninety-five percent confidence intervals for ARs, ARDs, and NNH were estimated using nonparametric bootstrap resampling. Stratified analyses were conducted to examine potential effect modification by sex, age, BMI, waist circumference, antihypertensive medication use, and hs-CRP.

In addition, a series of sensitivity analyses was conducted. First, participants who developed the outcome within the first two years of follow-up were excluded to minimize potential reverse causation. Second, to account for the potential metabolic effects of cancer, participants with a cancer diagnosis at baseline were excluded. Participants receiving antihypertensive or lipid-lowering therapy at baseline were also excluded in separate analyses. Moreover, IFG evolution groups were reclassified according to the World Health Organization (WHO) definition of IFG to assess the robustness of the models (1). In addition, to assess the potential influence of missing covariate data and the use of multiple imputation, we conducted a complete-case analysis restricted to participants with complete data for all covariates included in the fully adjusted model. Cox proportional hazards models were subsequently repeated in this restricted cohort to further evaluate the robustness of the primary findings. To assess the robustness of our findings to alternative CKD definitions, we conducted additional sensitivity analysis. Among participants with repeated kidney measurements, CKD was defined as an eGFR <60 mL/min/1.73 m² and/or proteinuria meeting the prespecified threshold at two consecutive health examinations. Finally, competing risk regression (Fine–Gray model) was applied to account for the potential competing risk of death (25).

All statistical analyses were performed using SAS version 9.4 and R version 4.5.2. For the prespecified primary outcome, two-sided *P* values <0.05 were considered statistically significant. Because analyses of secondary outcomes, subgroup analyses, follow-up time-stratified analyses, and sensitivity analyses were exploratory and were not formally adjusted for multiple comparisons, their estimates and confidence intervals were interpreted descriptively, with emphasis on the magnitude, direction, and consistency of associations rather than on formal statistical significance.

## Results

### Baseline characteristics

Among the 43,073 participants (**Table 1****;** **Supplementary Table 3**), the mean age was 51.30 ± 11.55 years, and 77.20% were men. Compared with the sustained normal fasting glucose group, participants in the persistent IFG group were older, more likely to be men, and had a higher proportion of smokers. They also exhibited higher levels of fasting blood glucose, BMI, systolic and diastolic blood pressure, LDL-C, and hs-CRP, as well as a higher prevalence of hypertension and greater use of antihypertensive medications. However, they had lower proportions of current alcohol consumption and higher educational attainment, along with lower levels of eGFR and HDL-C. No significant differences were observed in physical activity or in the use of lipid-lowering medications.

**Table 1:** Baseline characteristic of 43,073 participants according to impaired FBG evolution.

	Total	Sustained normal FBG	Impaired FBG progression	Impaired FBG recovery	Persistent impaired FBG	P value
	(N = 43,073)	(N = 27,105, 62.93%)	(N = 7,832, 18.18%)	(N = 4,157, 9.65%)	(N = 3,979, 9.24%)	
**Age, y**	51.30 ± 11.55	50.58 ± 11.85	53.58 ± 11.22	50.36 ± 10.88	52.68 ± 10.00	<0.01
**Men, N (%)**	33,252 (77.20)	19,932 (73.50)	6,364 (81.30)	3,486 (83.90)	3,470 (87.20)	<0.01
**Drink, N (%)**	26,852 (62.30)	17,441 (64.30)	4,782 (61.10)	2,410 (58.00)	2,219 (55.80)	<0.01
**Smoke, N (%)**	17,742 (41.20)	10,834 (40.00)	3,226 (41.20)	1,868 (44.90)	1,814 (45.60)	<0.01
**Physical activity, N (%)**	6,091 (14.10)	3,806 (14.00)	1,147 (14.60)	583 (14.00)	555 (13.90)	0.57
**High school education or above, N (%)**	5,762 (13.40)	4,097 (15.10)	722 (9.22)	562 (13.50)	381 (9.58)	<0.01
**FBG, mmol/L**	5.24 ± 0.60	4.94 ± 0.40	5.96 ± 0.32	5.07 ± 0.37	6.09 ± 0.37	<0.01
**BMI, kg/m^2^**	24.85 ± 3.26	24.59 ± 3.24	25.32 ± 3.30	24.99 ± 3.21	25.54 ± 3.14	<0.01
**SBP, mmHg**	128.71 ± 18.24	126.50 ± 17.87	132.88 ± 18.13	129.51 ± 18.16	134.72 ± 18.25	<0.01
**DBP, mmHg**	83.74 ± 10.60	82.62 ± 10.51	85.72 ± 10.48	84.49 ± 10.56	86.69 ± 10.28	<0.01
**LDL-C, mmol/L**	2.59 ± 0.77	2.52 ± 0.77	2.70 ± 0.78	2.61 ± 0.72	2.77 ± 0.76	<0.01
**HDL-C, mmol/L**	1.58 ± 0.51	1.58 ± 0.52	1.59 ± 0.53	1.55 ± 0.47	1.57 ± 0.47	<0.01
**eGFR, mL/min/1.73 m²**	94.38 ± 15.93	94.77 ± 16.17	93.08 ± 15.33	94.97 ± 15.81	93.68 ± 15.40	<0.01
**hs-CRP, mg/L**	1.20 (0.60–2.80)	1.15 (0.60–2.70)	1.31 (0.68–3.00)	1.20 (0.63–2.70)	1.30 (0.70–2.90)	-
**Hypertension, N (%)**	17,566 (40.80)	9,832 (36.30)	3,840 (49.00)	1,800 (43.30)	2,094 (52.60)	<0.01
**Antihypertensive drugs, N (%)**	4,243 (9.85)	2,320 (8.56)	963 (12.30)	450 (10.80)	510 (12.80)	<0.01
**Lipid-lowering drugs,** **N (%)**	486 (1.13)	290 (1.07)	83 (1.06)	62 (1.49)	51 (1.28)	0.07

FBG, fasting blood glucose; BMI, body mass index; SBP, systolic blood pressure; DBP, diastolic blood pressure; LDL-C, low-density lipoprotein cholesterol; HDL-C, high-density lipoprotein cholesterol; eGFR, estimated glomerular filtration rate; hs-CRP, high-sensitivity C-reactive protein.

Bold values represent n (%).

### IFG evolution and chronic disease multimorbidity

**Figure 1:**
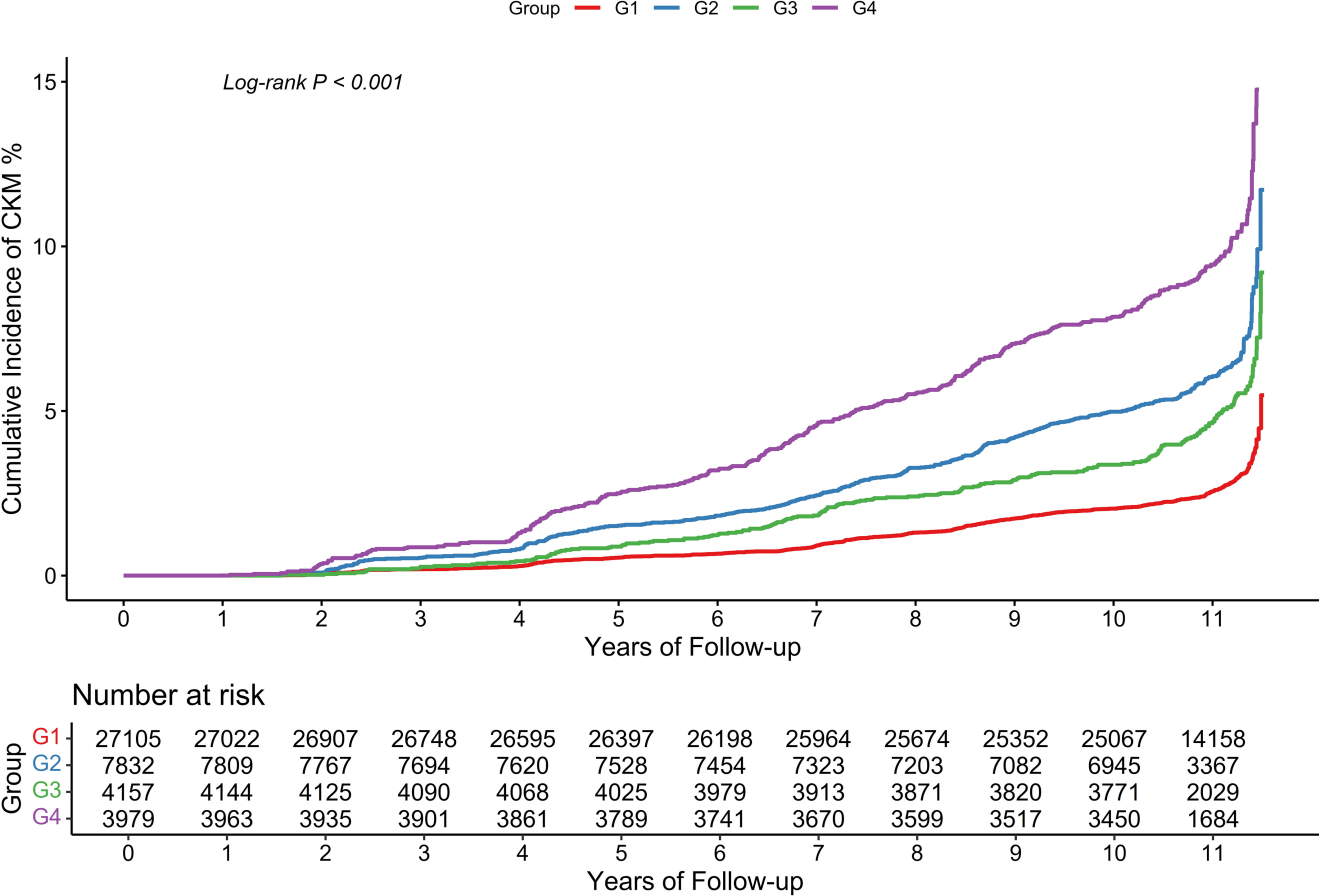
Cumulative incidence of cardio-kidney-metabolic disease multimorbidity by IFG progression. G1, Sustained Normal FBG; G2, Impaired FBG Progression; G3, Impaired FBG Recovery; G4, Persistent Impaired FBG.

During a median follow-up of 11.00 years (IQR: 10.62–11.32), a total of 1,795 cases of cardio-kidney-metabolic multimorbidity were identified. The cumulative incidence of multimorbidity according to impaired FBG evolution is shown in **Figure 1**. We also constructed a schematic disease-transition diagram to depict the progression from a disease-free state to single-disease and multimorbidity states (**Supplementary Figure 3**). Compared with the sustained normal fasting glucose group, the risk of developing cardio-kidney-metabolic multimorbidity increased by 93% in the IFG progression group (HR: 1.93; 95% CI: 1.72–2.17), by 69% in the IFG recovery group (HR: 1.69; 95% CI: 1.44–1.98), and by 210% in the persistent IFG group (HR: 3.10; 95% CI: 2.74–3.52) (**Table 2A****;** **Supplementary Table 4A**). Using the IFG recovery group as the reference, the sustained normal fasting glucose group had a 41% lower risk of cardio-kidney-metabolic multimorbidity (HR: 0.59; 95% CI: 0.51–0.69), whereas the IFG progression group had a 23% higher risk (HR: 1.14; 95% CI: 0.97–1.35) and the persistent IFG group had a 94% higher risk (HR: 1.84; 95% CI: 1.55–2.18) (**Table 2B****;** **Supplementary Table 4B**).

**Table 2:** HRs (95% CIs) for cardio-kidney-metabolic disease multimorbidity according to impaired fbg evolution.

A				
	Sustained normal FBG	Impaired FBG progression	Impaired FBG recovery	Persistent impaired FBG
Events/Total	737/27,105	479/7,832	198/4,157	381/3,979
IR, per 1,000 person-years	2.55	5.79	4.50	9.06
HR (95%CI)	1.00 (reference)	1.93 (1.72-2.17)	1.69 (1.44-1.98)	3.10 (2.74-3.52)
HR _year 0 to 5_ (95%CI)	1.00 (reference)	2.19 (1.71-2.80)	1.54 (1.07-2.22)	3.70 (2.85-4.80)
HR _year > 5_ (95%CI)	1.00 (reference)	1.86 (1.63-2.12)	1.72 (1.45-2.05)	2.94 (2.55-3.39)
B				
	Sustained normal FBG	Impaired FBG progression	Impaired FBG recovery	Persistent impaired FBG
Events/Total	737/27,105	479/7,832	198/4,157	381/3,979
IR, per 1,000 person-years	2.55	5.79	4.50	9.06
HR (95%CI)	0.59 (0.51-0.69)	1.14 (0.97-1.35)	1.00 (reference)	1.84 (1.55-2.18)
HR _year 0 to 5_ (95%CI)	0.65 (0.45-0.94)	1.42 (0.98-2.07)	1.00 (reference)	2.41 (1.64-3.53)
HR _year > 5_ (95%CI)	0.58 (0.49-0.69)	1.08 (0.90-1.30)	1.00 (reference)	1.70 (1.40-2.07)

(A) The sustained normal FBG group is used as the reference. (B) The impaired FBG recovery group is used as the reference.

Model 1 was an unadjusted crude model.

Model 2 was further adjusted for age, sex, education level, drink status, smoking status and physical activity.

Model 3 was further adjusted for body mass index, high-density lipoprotein cholesterol, low-density lipoprotein cholesterol and high- sensitivity C-reactive protein.

Model 4 was further adjusted for antihypertensive drugs and lipid-lowering drugs. HR, hazard ratio; FBG, fasting blood glucose; IR, incidence rate; CI, confidence interval.

Follow-up time-stratified analyses showed that the associations between impaired FBG evolution and cardio-kidney-metabolic multimorbidity were generally consistent across follow-up intervals. During the first 5 years of follow-up, compared with the sustained normal FBG group, the impaired FBG progression, impaired FBG recovery, and persistent impaired FBG groups were associated with higher risks of cardio-kidney-metabolic multimorbidity, with HRs of 2.19 (95% CI, 1.71–2.80), 1.54 (95% CI, 1.07–2.22), and 3.70 (95% CI, 2.85–4.80), respectively. Similar associations were observed during the follow-up period beyond 5 years. In the fully adjusted model, compared with the sustained normal FBG group, the impaired FBG progression, impaired FBG recovery, and persistent impaired FBG groups showed directionally consistent associations with higher risks of cardio-kidney-metabolic multimorbidity, with HRs of 1.86 (95% CI, 1.63–2.12), 1.72 (95% CI, 1.45–2.05), and 2.94 (95% CI, 2.55–3.39), respectively (**Table 2A**; **Supplementary Table 5A**). Moreover, when the impaired FBG recovery group was used as the reference, follow-up time-stratified analyses yielded patterns generally consistent with the main findings, showing a consistently lower risk among participants with sustained normal FBG and a higher risk among those with persistent impaired FBG across both follow-up intervals (**Table 2B**; **Supplementary Table 5B**). Overall, these follow-up time-stratified results suggest that the adverse associations between impaired FBG evolution and cardio-kidney-metabolic multimorbidity were not confined to the early follow-up period but persisted during longer-term follow-up.

To further quantify the clinical impact of different IFG evolution patterns, we estimated multivariable-adjusted AR, ARD, and the NNH (**Supplementary Table 6**). Compared with the sustained normal FBG group (AR: 2.25%; 95%CI: 1.28%-4.04%), the adjusted AR of cardio–kidney–metabolic multimorbidity was 4.28% in the IFG progression group, 3.76% in the IFG recovery group, and 6.74% in the persistent IFG group. The corresponding ARDs were 2.03% and 1.51% for the progression and recovery groups, respectively, and were highest in the persistent IFG group. The estimated NNH values were 49.26 for IFG progression, 66.23 for IFG recovery, and 22.27 for persistent IFG, indicating that approximately one additional case of multimorbidity would occur for every 23 individuals with persistent IFG compared with those maintaining sustained normal fasting glucose.

### IFG evolution and components of chronic disease multimorbidity

**Figure 2:**
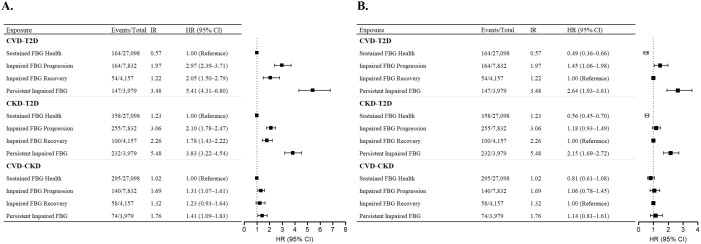
Association between impaired FBG progression and risk of CVD-T2D, CKD-T2D, and CVD-CKD multimorbidity. **(A)** Forest plot for single disease by the evolution of IFG status. The sustained fasting glucose health group is used as the reference. **(B)** Forest plot for single disease by the evolution of IFG status. The impaired FBG recovery group is used as the reference. Adjusted for age, sex, education level, drink status, smoking status, physical activity, body mass index, high-density lipoprotein cholesterol, low-density lipoprotein cholesterol, high- sensitivity C-reactive protein, antihypertensive drugs and lipid-lowering drugs. Abbreviations: FBG, fasting blood glucose; CVD, cardiovascular disease; T2D, type 2 diabetes; CKD, chronic kidney disease; IR, incidence rate/per 1,000 person-years; HR, hazard ratio; CI, confidence interval.

For the components of cardio-kidney-metabolic multimorbidity (**Figure 2**; **Supplementary Table 7**), the overall pattern of associations was broadly consistent with that observed in the primary analysis, particularly for T2D-related combinations. Compared with the sustained normal fasting glucose group, the IFG progression, IFG recovery, and persistent IFG groups showed higher HRs for CVD–T2D and CKD–T2D multimorbidity. The estimates for CVD–CKD multimorbidity were smaller in magnitude and less precise.

Follow-up time-stratified analyses for CVD–CKD multimorbidity showed generally consistent but attenuated associations compared with the overall analysis (**Table 3**; **Supplementary Table 8**). In the fully adjusted model, the confidence intervals included the null were observed during the first 5 years of follow-up. However, beyond 5 years, the impaired FBG progression and persistent impaired FBG groups were associated with higher risks of CVD–CKD multimorbidity. The impaired FBG recovery group was not significantly associated with CVD–CKD multimorbidity in either follow-up interval. When CVD–T2D and CKD–T2D multimorbidity were evaluated as outcomes, the observed associations were generally consistent with the main findings.

**Table 3:** HRs (95% CIs) for CVD–T2D, CKD–T2D, and CVD–CKD multimorbidity across follow-up time periods according to impaired FBG evolution.

A				
	Sustained normal FBG	Impaired FBG progression	Impaired FBG recovery	Persistent impaired FBG
CVD–T2D				
HR _year 0 to 5_ (95%CI)	1.00 (reference)	3.57 (2.04-6.26)	1.97 (0.84-4.62)	4.70 (2.52-8.75)
HR _year > 5_ (95%CI)	1.00 (reference)	2.88 (2.26-3.66)	2.06 (1.48-2.88)	5.56 (4.35-7.11)
CKD–T2D				
HR _year 0 to 5_ (95%CI)	1.00 (reference)	2.37 (1.68-3.32)	1.70 (1.04-2.79)	5.40 (3.87-7.52)
HR _year > 5_ (95%CI)	1.00 (reference)	2.01 (1.67-2.42)	1.79 (1.40-2.30)	3.40 (2.78-4.15)
CVD–CKD				
HR _year 0 to 5_ (95%CI)	1.00 (reference)	1.39 (0.90-2.16)	0.94 (0.46-1.90)	0.96 (0.50-1.84)
HR _year > 5_ (95%CI)	1.00 (reference)	1.29 (1.03-1.63)	1.31 (0.96-1.79)	1.53 (1.16-2.03)
B				
	Sustained normal FBG	Impaired FBG progression	Impaired FBG recovery	Persistent impaired FBG
CVD–T2D				
HR _year 0 to 5_ (95%CI)	0.50 (0.21-1.19)	1.81 (0.79-4.16)	1.00 (reference)	2.39 (1.00-5.70)
HR _year > 5_ (95%CI)	0.48 (0.35-0.68)	1.40 (1.00-1.95)	1.00 (reference)	2.70 (1.93-3.78)
CKD–T2D				
HR _year 0 to 5_ (95%CI)	0.59 (0.36-0.96)	1.39 (0.84-2.31)	1.00 (reference)	3.17 (1.93-5.23)
HR _year > 5_ (95%CI)	0.56 (0.43-0.71)	1.12 (0.86-1.46)	1.00 (reference)	1.89 (1.44-2.49)
CVD–CKD				
HR _year 0 to 5_ (95%CI)	1.07 (0.53-2.15)	1.48 (0.70-3.12)	1.00 (reference)	1.02 (0.42-2.47)
HR _year > 5_ (95%CI)	0.76 (0.56-1.04)	0.99 (0.70-1.39)	1.00 (reference)	1.17 (0.80-1.70)

(A) The sustained normal FBG group is used as the reference. (B) The impaired FBG recovery group is used as the reference.

Adjusted for age, sex, education level, drink status, smoking status, physical activity, body mass index, high-density lipoprotein cholesterol, low-density lipoprotein cholesterol, high- sensitivity C-reactive protein, antihypertensive drugs and lipid-lowering drugs.

HR, hazard ratio; CVD, cardiovascular disease; T2D, type 2 diabetes; CKD, chronic kidney disease; FBG, fasting blood glucose; CI, confidence interval.

**Figure 3:**
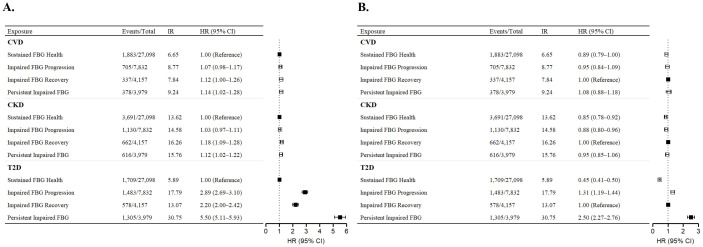
Association between impaired FBG progression and risk of cardiovascular disease, chronic kidney disease and type 2 diabetes. **(A)** Forest plot for single disease by the evolution of IFG status. The sustained fasting glucose health group is used as the reference. **(B)** Forest plot for single disease by the evolution of IFG status. The impaired FBG recovery group is used as the reference. Adjusted for age, sex, education level, drink status, smoking status, physical activity, body mass index, high-density lipoprotein cholesterol, low-density lipoprotein cholesterol, high- sensitivity C-reactive protein, antihypertensive drugs and lipid-lowering drugs. FBG, fasting blood glucose; CVD, cardiovascular disease; CKD, chronic kidney disease; T2D, type 2 diabetes; IR, incidence rate/ per 1,000 person-years; HR, hazard ratio; CI, confidence interval.

For single diseases, a total of 3,303 cases of CVD, 6,099 cases of CKD, and 5,075 cases of type 2 diabetes were recorded during the follow-up period. We further analyzed the associations between the different IFG evolution groups and each individual disease (**Figure 3**; **Supplementary Tables 9**) to support our conclusions. Notably, the apparently higher incidence of CKD observed in the IFG recovery group warrants cautious interpretation, as reductions in eGFR following glycemic improvement may partly represent the resolution of antecedent glomerular hyperfiltration rather than true progressive structural renal injury.

Follow-up time-stratified analyses for incident CVD showed broadly consistent but weaker associations than those observed for the primary CKM multimorbidity outcome (**Supplementary Tables 10**). Increases in CVD risk was mainly observed for the impaired FBG progression group during the first 5 years of follow-up and for the impaired FBG recovery and persistent impaired FBG groups beyond 5 years, although the overall effect sizes were modest. When CKD and type 2 diabetes were evaluated as single outcomes, the observed associations were generally consistent with the main findings (**Supplementary Tables 10**).

### Stratified and sensitivity analyses

In the stratified analyses, nominal evidence of interaction was observed for the use of antihypertensive medication (*P* for interaction < 0.01). Among participants using antihypertensive agents, the statistically significant differences were not observed between IFG progression and IFG recovery group, whereas the trend among those not receiving antihypertensive therapy was consistent with the main findings. This attenuated risk may reflect intensified management of cardiometabolic risk factors following the detection of metabolic abnormalities, as well as reduced statistical precision resulting from smaller sample sizes within stratified analyses (**Supplementary Tables 11**). Sensitivity analyses yielded estimates that were generally consistent with the main analysis in both direction and magnitude (**Supplementary Tables 12**). Notably, in the sensitivity analysis using a stricter CKD definition requiring abnormalities at two consecutive health examinations, the associations remained robust and directionally consistent with the primary analysis. The Sankey diagram illustrates that, after reclassification using the WHO criteria, most participants were reassigned to the sustained normal FBG group (**Supplementary Figure 4**). This pattern suggests that the fasting glucose elevations in this subset were predominantly mild. In the Fine–Gray competing risk model, the associations also remained robust (**Supplementary Table 12**).

## Discussion

In this large prospective cohort study, our findings demonstrate that all patterns of IFG evolution were significantly and positively associated with the risk of incident cardio-kidney-metabolic multimorbidity. Individuals with persistent IFG exhibited the highest risk, with the magnitude of risk increasing markedly as the duration of fasting glucose abnormality increased. In contrast, individuals who recovered from IFG or progressed to IFG had substantially lower risks than those with persistent IFG. To account for potential differences arising from alternative classification criteria, we conducted a sensitivity analysis using the WHO definition of IFG, which yielded similar results. These findings suggest that the observed association was not primarily driven by individuals with mildly elevated fasting glucose levels. The associations also remained largely consistent across additional sensitivity analyses.

To our knowledge, this is the first large-scale cohort study to examine the long-term impact of different IFG evolution patterns on the risk of incident cardio-kidney-metabolic multimorbidity. The adverse health consequences associated with early cumulative exposure to IFG, as well as the benefits of reversing IFG status, are consistent with previous evidence. A large cohort study from China demonstrated that reversion from IFG to normoglycemia was associated with reduced risks of future cardiovascular disease and all-cause mortality (26). Conversely, the presence of IFG has been shown to increase the risks of atrial fibrillation and heart failure in a dose–response manner (27). In addition, another study reported that IFG variability and cumulative glycemic exposure were strongly associated with the development of chronic kidney disease (28). However, these prior studies largely relied on single time-point assessments. Although some investigations have characterized IFG trajectories in relation to specific outcomes (14, 17), they have not comprehensively delineated the pathological evolution of IFG or examined its implications across multiple organ systems. Moreover, evidence suggests that not all glycemic trajectory patterns inevitably progress to diabetes (29), underscoring the importance of understanding the transition dynamics of IFG. Recent trajectory-based analyses in large population cohorts have further demonstrated that glycemic patterns often follow relatively stable long-term trajectories, suggesting that classification based on limited time points may still capture biologically meaningful exposure despite potential interval censoring (29, 30). Nevertheless, we acknowledge that classification based on two discrete fasting blood glucose measurements cannot fully reconstruct individual glycemic trajectories and may be subject to interval censoring and exposure misclassification. Therefore, our IFG evolution categories should be interpreted as clinically pragmatic approximations of glycemic history rather than complete representations of intra-individual glucose dynamics. In our large-sample study, which included two reliable fasting glucose measurements obtained four years apart and extended follow-up thereafter, we found that participants with a history of IFG had a lower risk of multimorbidity than those with persistent IFG.

When the IFG recovery group was used as the reference, the sustained normal fasting glucose group was associated with a substantially lower risk of CKM multimorbidity, whereas the persistent IFG group remained associated with a higher risk. The association for the IFG progression group was attenuated and no longer reached statistical significance in the fully adjusted model. These findings suggest that the optimal strategy is not merely achieving recovery after hyperglycemia emerges, but rather maintaining long-term fasting glucose health from the outset. Previous studies have similarly shown that even fasting glucose levels within the normal range follow a J-shaped risk pattern (10), whereby slight elevations substantially increase the likelihood of future diabetes and cardiovascular disease (15). In addition, prior dysglycemic exposure may be associated with residual vascular and renal risks, even after apparent normalization of fasting glucose (31). While HRs provide important information on the strength of association, AR metrics may offer a more clinically interpretable estimate of the population burden associated with different IFG evolution patterns. In the present study, individuals with IFG recovery, progression, and persistent IFG exhibited progressively higher ARs of cardio–kidney–metabolic multimorbidity compared with those maintaining sustained normal fasting glucose. These findings provide a clinically intuitive perspective on the magnitude of excess risk associated with different IFG evolution patterns. Notably, persistent IFG was associated with the greatest absolute burden of multimorbidity, suggesting that sustained dysglycemia may impose cumulative metabolic stress across multiple organ systems. Importantly, although individuals who recovered from IFG exhibited lower risk than those with persistent IFG, their absolute risk remained higher than that of individuals maintaining sustained normal fasting glucose, reinforcing the clinical observation that early metabolic disturbances may be associated with residual excess risk, even after apparent normalization of fasting glucose (32).

Taken together, these lines of evidence indicate that intervening before fasting glucose becomes abnormal is essential for maximizing long-term benefits and mitigating the accumulation of excess long-term risk. Previous studies suggest that remission from prediabetes may not fully restore metabolic risk to the level observed in individuals with sustained normoglycemia, supporting the clinical relevance of distinguishing IFG recovery from sustained normal fasting glucose (33). Collectively, the evidence underscores that maintaining sustained normal fasting glucose is not only the foundational approach to reducing the future burden of multimorbidity but also the most cost-effective strategy for mitigating long-term risks associated with metabolic dysfunction (18).

Notably, we also observed that the risk patterns associated with IFG progression and recovery were not consistent across different disease outcomes. For instance, when CKD was evaluated as an individual outcome, the risk in the IFG recovery group was substantially higher than that in the IFG progression group. However, for CKD-related composite outcomes, the IFG progression group paradoxically demonstrated a higher risk. Some studies suggest that this phenomenon may reflect an apparent decline in kidney function driven by the resolution of glomerular hyperfiltration rather than true renal deterioration. During the early stages of rising blood glucose, the kidney may induce dilation of the afferent arteriole or glomerular capillaries through specific pathways (34) or mediators (35), thereby leading to hyperfiltration. Similarly, when glycemic control is achieved with SGLT2 inhibitors, GFR decreases as hyperfiltration subsides (22). The resulting decline in filtration rate may therefore represent a physiological correction rather than true deterioration of renal function. This pseudo-decline may partly explain why the IFG recovery group demonstrated a higher risk than the IFG progression group.

This apparent discrepancy also suggests that the risks associated with fasting glucose recovery may vary across outcome types. The evolution of IFG may exert organ-specific and time-dependent effects across different physiological systems. In other words, although metabolic recovery may reduce systemic inflammation and cardiovascular risk, renal outcomes may be less reversible and more difficult to interpret when defined primarily by eGFR-based criteria (18, 22). The higher incidence of CKD observed in the IFG recovery group should be interpreted with caution. One possible explanation is that improvement in glycemic status may be accompanied by the resolution of antecedent glomerular hyperfiltration, leading to an apparent decline in eGFR rather than true structural renal deterioration (31, 36). These findings highlight the importance of early identification and intervention for dysglycemia before overt organ damage becomes clinically apparent.

In subgroup analyses, the associations between IFG evolution patterns and CKM multimorbidity were broadly consistent across most strata. A statistically significant interaction was observed for antihypertensive medication use, with an attenuated association between IFG progression and CKM multimorbidity among participants receiving antihypertensive therapy. This pattern may reflect intensified cardiometabolic risk management following the detection of metabolic abnormalities in this higher-risk group. However, stratification reduced the number of events within each subgroup, resulting in lower statistical precision and wider confidence intervals. These findings should therefore be interpreted cautiously and do not materially alter the overall conclusions of the primary analyses.

Although participants with IFG recovery had a lower risk of CKM multimorbidity than those with persistent IFG, their risk did not return to that observed among participants who maintained sustained normal fasting glucose. This finding should be interpreted primarily as clinical and epidemiological evidence that a history of IFG may identify individuals with persistent vulnerability to adverse CKM outcomes. The residual excess risk may reflect cumulative glycemic exposure before recovery, persistent clustering of cardiometabolic risk factors, and unmeasured lifestyle or metabolic influences. From a biological perspective, persistent or recurrent dysglycemia may promote low-grade inflammation, oxidative stress, endothelial dysfunction, microvascular injury, and neurohormonal activation, thereby contributing to a shared pathological substrate for cardiac and renal injury (37, 38). In the kidney, dysglycemia-related microvascular injury and increased tubular energy demand may induce local hypoxia and metabolic reprogramming in renal tubular cells, thereby contributing to tubular injury, interstitial inflammation, and fibrotic remodeling (39, 40). Kidney fibrosis, characterized by maladaptive repair and excessive extracellular matrix deposition, represents a common final pathway of chronic kidney injury and may interact bidirectionally with cardiovascular injury through inflammation, vascular stiffness, volume overload, and metabolic dysregulation (41). Therefore, the persistent excess risk observed after IFG recovery may reflect not only current glycemic status but also prior cumulative glycemic exposure and residual multi-organ vulnerability. This interpretation is consistent with prognostic approaches that use longitudinal metabolic and renal profiles to identify individuals at increased risk of renal progression and cardiovascular events. This interpretation is consistent with real-world prognostic studies of diabetic complications and extends this framework to earlier dysglycemia, suggesting that a history of IFG before overt diabetes may help identify individuals susceptible to future CKM multimorbidity (42). However, because molecular biomarkers, endothelial function markers, renal fibrosis markers, and omics data were not available in the present study, these mechanistic explanations should be regarded as hypothesis-generating and require validation in future studies incorporating dedicated biomarker and mechanistic assessments.

Our findings indicate that dynamic monitoring of fasting glucose levels in community populations may serve as an early tool for predicting the risk of cardio-kidney-metabolic multimorbidity. In recent years, multiple studies have demonstrated that lifestyle interventions, weight management, and comprehensive control of multiple risk factors can markedly reduce the incidence of cardio-kidney-metabolic events. For example, the SELECT trial showed that in individuals with obesity but without diabetes, once-weekly semaglutide 2.4 mg significantly reduced the risk of major adverse cardiovascular events (MACE) (43). The FLOW trial further demonstrated that the GLP-1 receptor agonist semaglutide provided substantial cardio-renal protection in patients with type 2 diabetes and chronic kidney disease (44). Moreover, compared with the general population, individuals with chronic kidney disease whose blood pressure, lipid levels, and glycemic status are well controlled do not exhibit an increased risk of myocardial infarction or stroke (21). Consistently, the 2023 AHA scientific statement (18) and the 2025 ADA Standards of Care (45) both emphasize that dynamic glucose monitoring, weight management, and combination pharmacologic interventions constitute core strategies for preventing and managing cardio-kidney-metabolic syndrome.

### Strengths and limitations

The strengths of this study include its large sample size, stable community-based cohort, long follow-up duration, repeated fasting glucose measurements, and comprehensive covariate information. The clinically interpretable IFG transition framework allowed us to distinguish sustained normal fasting glucose from IFG progression, recovery, and persistence. Several limitations should also be noted. First, the Kailuan cohort consisted predominantly of men from northern China, which may limit the generalizability of our findings to women and to other geographic or ethnic populations. Second, IFG evolution was defined using fasting blood glucose measurements obtained at the 2006 and 2010 examinations. Although this two-point framework is clinically interpretable and reflects a pragmatic approach for population-based risk stratification, it may have introduced interval censoring and exposure misclassification. Because FBG was not repeatedly measured between the two examinations, the exact timing of IFG onset, remission, or recurrence could not be determined. If longer and more sustained dysglycemic exposure confers greater cardio-kidney-metabolic risk, this type of exposure misclassification would most plausibly attenuate the estimated association for persistent IFG toward the null, leading to potential underestimation rather than overestimation of the true risk. Nevertheless, the exact magnitude, and possibly the direction for some transition categories, could not be fully quantified in the absence of additional fasting glucose measurements. Moreover, intermediate glycemic fluctuations, glycemic re-progression after 2010, and abnormalities detectable only by HbA1c or oral glucose tolerance testing could not be captured (46, 47). Future studies incorporating more frequent fasting glucose assessments, HbA1c, oral glucose tolerance testing, or continuous glucose monitoring are warranted to more accurately characterize glycemic trajectories and cumulative glycemic exposure. Third, CKD was identified using creatinine-based eGFR and urine dipstick proteinuria, without cystatin C, quantitative albuminuria, or repeated confirmatory testing over at least 3 months; however, sensitivity analyses using stricter CKD definitions yielded generally consistent results. Finally, residual confounding remains possible, and analyses of secondary outcomes, subgroup effects, follow-up time strata, and sensitivity analyses were exploratory and were not formally adjusted for multiple comparisons.

## Conclusion

Our findings indicate that even when current fasting glucose levels fall within the normal range, any prior episode of IFG remains associated with an elevated risk of cardio-kidney-metabolic multimorbidity. Furthermore, our findings suggest that individuals with a history of IFG may benefit from organ- and system-specific assessments for potential damage, together with long-term monitoring, particularly for organs in which damage may be less reversible or more difficult to detect at an early stage.

## Data Availability

The original contributions presented in the study are included in the article/**Supplementary Material**. Further inquiries can be directed to the corresponding authors.
