# Supplementary material for: Impact of the longitudinal evolution of impaired fasting glucose on cardio-kidney-metabolic multimorbidity
Source: Front Endocrinol (Lausanne). 2026 Jun 16;17:1812122. doi: 10.3389/fendo.2026.1812122 (PMC13314427; doi:10.3389/fendo.2026.1812122)
Supplement: Supplementary file 1 [file DataSheet1.doc]

**Supplemental Material**

**Supplemental Material**

**Supplemental Tables**

**Supplemental Table 1.** Proportional Hazards Assumption Test for Impaired FBG Evolution Groups

**Supplemental Table 2.** Missing Covariates

**Supplemental Table 3.** Baseline Characteristic of 43,073 Participants According to Impaired FBG Evolution in 2006

**Supplemental Table 4.** HRs (95% CIs) for Cardio-Kidney-Metabolic Disease Multimorbidity According to Impaired FBG Evolution

**Supplemental Table 5**. HRs (95% CIs) Stratified by Follow-up Time Periods for Cardio-Kidney-Metabolic Disease Multimorbidity According to Impaired FBG Evolution

**Supplemental Table 6.** Adjusted Ten-Year ARs (95% CIs) for Cardio-Kidney-Metabolic Multimorbidity and Secondary Outcomes According to Impaired FBG Evolution

**Supplemental Table 7.** Association Between Impaired FBG Progression and Risk of CVD-T2D, CKD-T2D, and CVD-CKD Multimorbidity

**Supplemental Table 8.** Association Between Impaired FBG Progression and Risk of CVD-T2D, CKD-T2D, and CVD-CKD Multimorbidity Stratified by Follow-up Time Periods

**Supplemental Table 9.** Association Between Impaired FBG Progression and Risk of Cardiovascular Disease, Chronic Kidney Disease and Type 2 Diabetes

**Supplemental Table 10.** Association Between Impaired FBG Progression and Risk of Cardiovascular Disease, Chronic Kidney Disease and Type 2 Diabetes Stratified by Follow-up Time Periods

**Supplemental Table 11.** Stratified Analysis

**Supplemental Table 12.** Sensitivity Analysis

**Supplemental Table 13.** Fine-Gray

**Supplemental Figures**

**Supplemental Figure 1.** Study Flowchart

**Supplemental Figure 2.** Restricted Cubic Spline for Fasting Blood Glucose in 2010

**Supplemental Figure 3.** Multi-State Transition Patterns from Baseline to Cardio-Kidney-Metabolic Multimorbidity

**Supplemental Figure 4.** Visualization of Transition Patterns of IFG Progression under Different Classification Criteria

**Supplemental Table 1. Proportional Hazards Assumption Test for Impaired FBG Evolution Groups**

| **Exposure group** | **χ²** | ***P* value** | **Interpretation** |
| --- | --- | --- | --- |
| **Sustained Normal FBG** | Reference | - | - |
| **Impaired FBG Progression** | 0.742 | 0.389 | No evidence of PH violation |
| **Impaired FBG Recovery** | 0.398 | 0.528 | No evidence of PH violation |
| **Persistent Impaired FBG** | 1.562 | 0.211 | No evidence of PH violation |

Abbreviations: FBG: fasting blood glucose; PH, proportional hazards.

| **Supplemental Table 2. Missing Covariates** | | |
| --- | --- | --- |
| **Covariate** | **Number of Missing Values** | **Percentage of Missing Values** |
| **Drinking status** | 303 | 0.70% |
| **Smoking status** | 182 | 0.42% |
| **Physical activity** | 185 | 0.43% |
| **hs-CRP** | 2,974 | 6.90% |
| **BMI** | 1,370 | 3.18% |
| **Systolic blood pressure** | 1,056 | 2.45% |
| **HDL-C** | 8 | 0.02% |
| **LDL-C** | 104 | 0.24% |
| **eGFR** | 85 | 0.20% |

Abbreviations: hs-CRP, High-Sensitivity C-Reactive Protein; BMI, body mass index; HDL-C, high density lipoprotein cholesterol; LDL-C, low density lipoprotein cholesterol; GFR, estimated glomerular filtration rate.

**Supplemental Table 3. Baseline Characteristic of 43,073 Participants According to Impaired FBG Evolution in 2006**

|  | **Total** | **Sustained Normal FBG** | **Impaired FBG Progression** | **Impaired FBG Recovery** | **Persistent Impaired FBG** | **P Value** |
| --- | --- | --- | --- | --- | --- | --- |
|  | **(N=43,073)** | **(N=27,105, 62.93%)** | **(N=7,832, 18.18%)** | **(N=4,157, 9.65%)** | **(N=3,979, 9.24%)** |  |
| **Age, y** | 47.27±11.42 | 46.57±11.72 | 49.50±11.06 | 46.35±10.79 | 48.62±9.86 | <0.01 |
| **Men, N (%)** | 33,252 (77.20) | 19,932 (73.50) | 6,364 (81.30) | 3,486 (83.90) | 3,470 (87.20) | <0.01 |
| **Drink, N (%)** | 19,179 (44.50) | 11,449 (42.20) | 3,454 (44.10) | 2,155 (51.80) | 2,121 (53.30) | <0.01 |
| **Smoke, N (%)** | 17,891 (41.50) | 10,738 (39.60) | 3,240 (41.40) | 1,958 (47.10) | 1,955 (49.10) | <0.01 |
| **Physical activity, N (%)** | 5,331 (12.40) | 3,295 (12.20) | 1,024 (13.10) | 509 (12.20) | 503 (12.60) | 0.17 |
| **High school education or above, N (%)** | 4,066 (9.44) | 2,905 (10.70) | 448 (5.72) | 411 (9.89) | 302 (7.59) | <0.01 |
| **FBG, mmol/L** | 5.02±0.65 | 4.76±0.48 | 4.96±0.45 | 5.95±0.31 | 6.01±0.34 | <0.01 |
| **BMI, kg/m^2^** | 24.69±3.35 | 24.40±3.36 | 25.03±3.30 | 25.00±3.28 | 25.64±3.20 | <0.01 |
| **SBP, mmHg** | 125.59±18.68 | 123.76±18.25 | 128.31±18.51 | 127.38±19.27 | 130.84±19.41 | <0.01 |
| **DBP, mmHg** | 81.45±11.02 | 80.53±10.85 | 82.54±10.90 | 82.61±11.34 | 84.40±11.28 | <0.01 |
| **LDL-C, mmol/L** | 2.23±0.82 | 2.16±0.81 | 2.24±0.81 | 2.46±0.82 | 2.43±0.79 | <0.01 |
| **HDL-C, mmol/L** | 1.54±0.39 | 1.55±0.38 | 1.54±0.41 | 1.52±0.37 | 1.52±0.37 | <0.01 |
| **eGFR, mL/min/1.73 m²** | 88.54±17.76 | 87.82±17.49 | 88.49±17.34 | 91.32±19.73 | 90.64±17.79 | <0.01 |
| **hs-CRP, mg/L** | 0.70 (0.26–1.90) | 0.67 (0.24–1.90) | 0.73 (0.30–1.89) | 0.70 (0.29–1.90) | 0.78 (0.30–1.89) | - |
| **Hypertension, N (%)** | 14,724 (34.20) | 8,275 (30.50) | 3,049 (38.90) | 1,611 (38.80) | 1,789 (45.00) | <0.01 |
| **Antihypertensive drugs, N (%)** | 3,798 (8.82) | 2,119 (7.82) | 850 (10.90) | 393 (9.45) | 436 (11.00) | <0.01 |
| **Lipid-lowering drugs,**  **N (%)** | 716 (1.66) | 409 (1.51) | 158 (2.02) | 58 (1.40) | 91 (2.29) | <0.01 |
| Abbreviations: FBG, fasting blood glucose; BMI, body mass index; SBP, systolic blood pressure; DBP, diastolic pressure; LDL-C, low-density lipoprotein cholesterol; HDL-C, high-density lipoprotein cholesterol; eGFR, estimated glomerular filtration rate; hs-CRP, high-sensitivity C-reactive protein. | | | | | | |

| **Supplemental Table 4. HRs (95% CIs) for Cardio-Kidney-Metabolic Disease Multimorbidity According to Impaired FBG Evolution**  **A** | | | | |
| --- | --- | --- | --- | --- |
|  | **Sustained Normal FBG** | **Impaired FBG Progression** | **Impaired FBG Recovery** | **Persistent Impaired FBG** |
| Events/Total | 737/27,105 | 479/7,832 | 198/4,157 | 381/3,979 |
| IR, per 1,000 person-years | 2.55 | 5.79 | 4.50 | 9.06 |
| Model 1 | 1.00 (reference) | 2.39 (2.13-2.68) | 1.78 (1.53-2.29) | 3.82 (3.16-4.32) |
| Model 2 | 1.00 (reference) | 2.14 (1.91-2.40) | 1.78 (1.52-2.08) | 3.52 (3.11-3.99) |
| Model 3 | 1.00 (reference) | 1.95 (1.74-2.19) | 1.71 (1.46-2.00) | 3.16 (2.78-3.58) |
| Model 4 | 1.00 (reference) | 1.93 (1.72-2.17) | 1.69 (1.44-1.98) | 3.10 (2.74-3.52) |
| **B** | | | | |
|  | **Sustained Normal FBG** | **Impaired FBG Progression** | **Impaired FBG Recovery** | **Persistent Impaired FBG** |
| Events/Total | 737/27,105 | 479/7,832 | 198/4,157 | 381/3,979 |
| IR, per 1,000 person-years | 2.55 | 5.79 | 4.50 | 9.06 |
| Model 1 | 0.56 (0.48-0.66) | 1.34 (1.13-1.58) | 1.00 (reference) | 2.14 (1.80-2.54) |
| Model 2 | 0.56 (0.48-0.66) | 1.21 (1.02-1.42) | 1.00 (reference) | 1.98 (1.67-2.36) |
| Model 3 | 0.58 (0.50-0.68) | 1.14 (0.96-1.35) | 1.00 (reference) | 1.84 (1.55-2.19) |
| Model 4 | 0.59 (0.51-0.69) | 1.14 (0.97-1.35) | 1.00 (reference) | 1.84 (1.55-2.18) |
| (A) The sustained normal FBG group is used as the reference. (B) The impaired FBG recovery group is used as the reference. | | | | |
| Model 1 was an unadjusted crude model. | | | | |
| Model 2 was further adjusted for age, sex, education level, drink status, smoking status and physical activity. | | | | |
| Model 3 was further adjusted for body mass index, high-density lipoprotein cholesterol, low-density lipoprotein cholesterol and high- sensitivity C-reactive protein. | | | | |
| Model 4 was further adjusted for antihypertensive drugs and lipid-lowering drugs. | | | | |
| Abbreviations: HR, hazard ratio; FBG, fasting blood glucose; IR, incidence rate. | | | | |

| **Supplemental Table 5. HRs (95% CIs) Stratified by Follow-up Time Periods for Cardio-Kidney-Metabolic Disease Multimorbidity According to Impaired FBG Evolution**  **A.** | | | | |
| --- | --- | --- | --- | --- |
|  | **Sustained Normal FBG** | **Impaired FBG Progression** | **Impaired FBG Recovery** | **Persistent Impaired FBG** |
| **Follow-up Time from 0 to 5 years** | | | | |
| Events/Total | 146/27,105 | 117/7,832 | 36/4,157 | 98/3,979 |
| IR, per 1,000 person-years | 1.09 | 3.03 | 1.75 | 5.01 |
| Model 1 | 1.00 (reference) | 2.79 (2.19-3.56) | 1.61 (1.12-2.32) | 4.62 (3.58-5.97) |
| Model 2 | 1.00 (reference) | 2.46 (1.93-3.15) | 1.64 (1.14-2.37) | 4.30 (3.33-5.57) |
| Model 3 | 1.00 (reference) | 2.21 (1.73-2.83) | 1.57 (1.09-2.26) | 3.77 (2.91-4.90) |
| Model 4 | 1.00 (reference) | 2.19 (1.71-2.80) | 1.54 (1.07-2.22) | 3.70 (2.85-4.80) |
| **Follow-up Time > 5years** | | | | |
| Events/Total | 591/26,397 | 362/7,528 | 162/4,025 | 283/3,789 |
| IR, per 1,000 person-years | 3.84 | 8.41 | 7.03 | 13.20 |
| Model 1 | 1.00 (reference) | 2.28 (2.00-2.60) | 1.83 (1.54-2.18) | 3.61 (3.13-4.16) |
| Model 2 | 1.00 (reference) | 2.05 (1.80-2.35) | 1.81 (1.52-2.15) | 3.32 (2.88-3.83) |
| Model 3 | 1.00 (reference) | 1.88 (1.65-2.15) | 1.74 (1.46-2.07) | 2.98 (2.58-3.45) |
| Model 4 | 1.00 (reference) | 1.86 (1.63-2.12) | 1.72 (1.45-2.05) | 2.94 (2.55-3.39) |
| **B.** | | | | |
|  | **Sustained Normal FBG** | **Impaired FBG Progression** | **Impaired FBG Recovery** | **Persistent Impaired FBG** |
| **Follow-up Time from 0 to 5 years** | | | | |
| Events/Total | 146/27,105 | 117/7,832 | 36/4,157 | 98/3,979 |
| IR, per 1,000 person-years | 1.09 | 3.03 | 1.75 | 5.01 |
| Model 1 | 0.62 (0.43-0.89) | 1.73 (1.19-2.52) | 1.00 (reference) | 2.87 (1.96-4.20) |
| Model 2 | 0.61 (0.42-0.88) | 1.50 (1.03-2.18) | 1.00 (reference) | 2.62 (1.79-3.84) |
| Model 3 | 0.64 (0.44-0.92) | 1.41 (0.97-2.05) | 1.00 (reference) | 2.40 (1.64-3.53) |
| Model 4 | 0.65 (0.45-0.94) | 1.42 (0.98-2.07) | 1.00 (reference) | 2.41 (1.64-3.53) |
| **Follow-up Time > 5years** | | | | |
| Events/Total | 591/26,397 | 362/7,528 | 162/4,025 | 283/3,789 |
| IR, per 1,000 person-years | 3.84 | 8.41 | 7.03 | 13.20 |
| Model 1 | 0.55 (0.46-0.65) | 1.25 (1.04-1.50) | 1.00 (reference) | 1.98 (1.63-2.40) |
| Model 2 | 0.55 (0.46-0.66) | 1.14 (0.94-1.37) | 1.00 (reference) | 1.84 (1.51-2.23) |
| Model 3 | 0.57 (0.48-0.68) | 1.08 (0.90-1.30) | 1.00 (reference) | 1.71 (1.41-2.08) |
| Model 4 | 0.58 (0.49-0.69) | 1.08 (0.90-1.30) | 1.00 (reference) | 1.70 (1.40-2.07) |
| (A) The sustained normal FBG group is used as the reference. (B) The impaired FBG recovery group is used as the reference. | | | | |
| Model 1 was an unadjusted crude model. | | | | |
| Model 2 was further adjusted for age, sex, education level, drink status, smoking status and physical activity. | | | | |
| Model 3 was further adjusted for body mass index, high-density lipoprotein cholesterol, low-density lipoprotein cholesterol and high- sensitivity C-reactive protein. | | | | |
| Model 4 was further adjusted for antihypertensive drugs and lipid-lowering drugs. | | | | |
| Abbreviations: HR: hazard ratio; CI, confidence interval; FBG: fasting blood glucose; IR: incidence rate. | | | | |

**Supplemental Table 6. Adjusted Ten-Year ARs (95% CIs) for Cardio-Kidney-Metabolic Multimorbidity and Secondary Outcomes According to Impaired FBG Evolution**

|  | **Sustained Normal FBG** | **Impaired FBG Progression** | **Impaired FBG Recovery** | **Persistent Impaired FBG** |
| --- | --- | --- | --- | --- |
| **CKM** |  |  |  |  |
| AR (95% CI)/% | 2.25 (1.28-4.04) | 4.28 (2.47-7.49) | 3.76 (2.11-6.82) | 6.74 (3.83-11.79) |
| ARD (95% CI)/% | Reference | 2.03 (1.13-3.59) | 1.51 (0.75-2.85) | 4.49 (2.53-7.72) |
| NNH (95% CI) | Reference | 49.26 (27.86-88.50) | 66.23 (35.09-133.33) | 22.27 (12.95-39.53) |
| **CVD-T2D** |  |  |  |  |
| AR (%)  (95% CI) | 0.43 (0.43-1.25) | 1.28 (0.46-3.67) | 0.88 (0.32-2.72) | 2.30 (0.84-6.58) |
| ARD (%)  (95% CI) | Reference | 0.84 (0.29-2.44) | 0.45 (0.14-1.52) | 1.87 (0.68-5.34) |
| NNH (95% CI) | Reference | 119.05 (40.98-344.83) | 222.22 (65.79-714.29) | 53.48 (18.73-147.06) |
| **CKD-T2D** |  |  |  |  |
| AR (%)  (95% CI) | 1.16 (0.53-2.62) | 2.42 (1.10-5.40) | 2.06 (0.91-4.69) | 4.35 (2.02-9.55) |
| ARD (%)  (95% CI) | Reference | 1.26 (0.55-2.82) | 0.90 (0.34-2.19) | 3.19 (1.46-6.99) |
| NNH (95% CI) | Reference | 79.37 (35.46-181.82) | 111.11 (45.66-294.12) | 31.35 (14.31-68.49) |
| **CVD-CKD** |  |  |  |  |
| AR (%)  (95% CI) | 0.90 (0.31-2.78) | 1.18 (0.42-3.49) | 1.11 (0.37-3.39) | 1.26 (0.41-3.80) |
| ARD (%)  (95% CI) | Reference | 0.28 (0.05-0.91) | 0.21 (-0.06-0.91) | 0.36 (0.06-1.26) |
| NNH (95% CI) | Reference | 357.14 (109.89-2000.00) | 476.19 (109.89-∞; CI crosses 0) | 277.78 (79.37-1666.67) |
| **CVD** |  |  |  |  |
| AR (%)  (95% CI) | 5.97 (3.97-8.77) | 6.36 (4.21-9.37) | 6.66 (4.39-9.90) | 6.76 (4.46-10.03) |
| ARD (%)  (95% CI) | Reference | 0.39 (-0.12-0.98) | 0.69 (0.01-1.53) | 0.79 (0.10-1.72) |
| NNH (95% CI) | Reference | 256.41 (102.04-∞; CI crosses 0) | 144.93 (65.36-10000.00) | 126.58 (58.14-1000.00) |
| **CKD** |  |  |  |  |
| AR (%)  (95% CI) | 12.97 (9.59-17.25) | 13.39 (9.91-17.87) | 15.12 (11.14-20.07) | 14.37 (10.69-19.08) |
| ARD (%)  (95% CI) | Reference | 0.41 (-0.44-1.28) | 2.15 (1.02-3.54) | 1.40 (0.30-2.62) |
| NNH (95% CI) | Reference | 243.90 (78.13-∞; CI crosses 0) | 46.51 (28.25-98.04) | 71.43 (38.17-333.33) |
| **T2D** |  |  |  |  |
| AR (%)  (95% CI) | 5.77 (4.16-8.04) | 15.61 (11.47-21.12) | 12.18 (8.73-16.87) | 27.30 (20.57-35.73) |
| ARD (%)  (95% CI) | Reference | 9.84 (7.23-13.15) | 6.41 (4.46-8.93) | 21.53 (16.34-27.83) |
| NNH (95% CI) | Reference | 10.16 (7.60-13.83) | 15.60 (11.20-22.42) | 4.64 (3.59-6.12) |
| Adjusted for age, sex, education level, drink status, smoking status, physical activity, body mass index, high-density lipoprotein cholesterol, low-density lipoprotein cholesterol, high- sensitivity C-reactive protein, antihypertensive drugs and lipid-lowering drugs | | | | |

Abbreviations: AR: absolute risk; FBG: fasting blood glucose; ARD: absolute risk difference; CKM: Cardio-Kidney-Metabolic; CVD: cardiovascular disease; T2D: type 2 diabetes; CKD: chronic kidney disease.

**Supplemental Table 7. Association Between Impaired FBG Progression and Risk of CVD-T2D, CKD-T2D, and CVD-CKD Multimorbidity**

| **A.** | | | | |
| --- | --- | --- | --- | --- |
| **CVD-T2D** | | | | |
|  | **Sustained Normal FBG** | **Impaired FBG Progression** | **Impaired FBG Recovery** | **Persistent Impaired FBG** |
| Events/Total | 164/27,098 | 164/7,832 | 54/4,157 | 147/3,979 |
| IR, per 1,000 person-years | 0.57 | 1.97 | 1.22 | 3.48 |
| Model 1 | 1.00 (reference) | 3.65 (2.94-4.54) | 2.18 (1.60-2.97) | 6.80 (5.44-8.51) |
| Model 2 | 1.00 (reference) | 3.29 (2.64-4.09) | 2.15 (1.58-2.93) | 6.18 (4.93-7.74) |
| Model 3 | 1.00 (reference) | 3.01 (2.41-3.74) | 2.06 (1.51-2.81) | 5.49 (4.37-6.90) |
| Model 4 | 1.00 (reference) | 2.97 (2.39-3.71) | 2.05 (1.50-2.79) | 5.41 (4.31-6.80) |

| **CKD-T2D** | | | | |
| --- | --- | --- | --- | --- |
|  | **Sustained Normal FBG** | **Impaired FBG Progression** | **Impaired FBG Recovery** | **Persistent Impaired FBG** |
| Events/Total | 358/27,098 | 255/7,832 | 100/4,157 | 232/3,979 |
| IR, per 1,000 person-years | 1.23 | 3.06 | 2.26 | 5.48 |
| Model 1 | 1.00 (reference) | 2.40 (2.04-2.82) | 1.83 (1.47-2.28) | 4.42 (3.74-5.22) |
| Model 2 | 1.00 (reference) | 2.24 (1.90-2.63) | 1.84 (1.48-2.30) | 4.26 (3.60-5.04) |
| Model 3 | 1.00 (reference) | 2.10 (1.78-2.47) | 1.79 (1.43-2.24) | 3.86 (3.25-4.58) |
| Model 4 | 1.00 (reference) | 2.10 (1.78-2.47) | 1.78 (1.43-2.22) | 3.83 (3.22-4.54) |

| **CVD-CKD** | | | | |
| --- | --- | --- | --- | --- |
|  | **Sustained Normal FBG** | **Impaired FBG Progression** | **Impaired FBG Recovery** | **Persistent Impaired FBG** |
| Events/Total | 295/27,098 | 140/7,832 | 58/4,157 | 74/3,979 |
| IR, per 1,000 person-years | 1.02 | 1.69 | 1.32 | 1.76 |
| Model 1 | 1.00 (reference) | 1.72 (1.41-2.10) | 1.27 (0.96-1.68) | 1.80 (1.39-2.32) |
| Model 2 | 1.00 (reference) | 1.47 (1.20-1.80) | 1.30 (0.98-1.72) | 1.63 (1.26-2.11) |
| Model 3 | 1.00 (reference) | 1.34 (1.09-1.64) | 1.25 (0.94-1.66) | 1.45 (1.12-1.88) |
| Model 4 | 1.00 (reference) | 1.31 (1.07-1.61) | 1.23 (0.93-1.64) | 1.41 (1.09-1.83) |

| **B.** | | | | |
| --- | --- | --- | --- | --- |
| **CVD-T2D** | | | | |
|  | **Sustained Normal FBG** | **Impaired FBG Progression** | **Impaired FBG Recovery** | **Persistent Impaired FBG** |
| Events/Total | 164/27,098 | 164/7,832 | 54/4,157 | 147/3,979 |
| IR, per 1,000 person-years | 0.57 | 1.97 | 1.22 | 3.48 |
| Model 1 | 0.46 (0.34-0.63) | 1.68 (1.23-2.28) | 1.00 (reference) | 3.12 (2.28-4.27) |
| Model 2 | 0.47 (0.34-0.63) | 1.53 (1.12-2.09) | 1.00 (reference) | 2.87 (2.10-3.93) |
| Model 3 | 0.48 (0.36-0.66) | 1.46 (1.07-1.99) | 1.00 (reference) | 2.66 (1.94-3.64) |
| Model 4 | 0.49 (0.36-0.66) | 1.45 (1.06-1.98) | 1.00 (reference) | 2.64 (1.93-3.61) |
|  |  |  |  |  |
| **CKD-T2D** | | | | |
|  | **Sustained Normal FBG** | **Impaired FBG Progression** | **Impaired FBG Recovery** | **Persistent Impaired FBG** |
| Events/Total | 358/27,098 | 255/7,832 | 100/4,157 | 232/3,979 |
| IR, per 1,000 person-years | 1.23 | 3.06 | 2.26 | 5.48 |
| Model 1 | 0.55 (0.44-0.68) | 1.31 (1.04-1.65) | 1.00 (reference) | 2.42 (1.91-3.05) |
| Model 2 | 0.54 (0.43-0.68) | 1.21 (0.96-1.53) | 1.00 (reference) | 2.31 (1.83-2.92) |
| Model 3 | 0.56 (0.45-0.70) | 1.17 (0.93-1.48) | 1.00 (reference) | 2.16 (1.70-2.73) |
| Model 4 | 0.56 (0.45-0.70) | 1.18 (0.93-1.49) | 1.00 (reference) | 2.15 (1.69-2.72) |

| **CVD-CKD** | | | | |
| --- | --- | --- | --- | --- |
|  | **Sustained Normal FBG** | **Impaired FBG Progression** | **Impaired FBG Recovery** | **Persistent Impaired FBG** |
| Events/Total | 295/27,098 | 140/7,832 | 58/4,157 | 74/3,979 |
| IR, per 1,000 person-years | 1.02 | 1.69 | 1.32 | 1.76 |
| Model 1 | 0.79 (0.60-1.05) | 1.36 (1.00-1.85) | 1.00 (reference) | 1.42 (1.01-2.00) |
| Model 2 | 0.77 (0.58-1.02) | 1.13 (0.83-1.54) | 1.00 (reference) | 1.26 (0.89-1.78) |
| Model 3 | 0.80 (0.60-1.06) | 1.07 (0.78-1.45) | 1.00 (reference) | 1.16 (0.82-1.64) |
| Model 4 | 0.81 (0.61-1.08) | 1.06 (0.78-1.45) | 1.00 (reference) | 1.14 (0.81-1.61) |
| (A) The sustained normal FBG group is used as the reference. (B) The impaired FBG recovery group is used as the reference. | | | | |
| Model 1 was an unadjusted crude model. | | | | |
| Model 2 was further adjusted for age, sex, education level, drink status, smoking status and physical activity. | | | | |
| Model 3 was further adjusted for body mass index, high-density lipoprotein cholesterol, low-density lipoprotein cholesterol and high- sensitivity C-reactive protein. | | | | |
| Model 4 was further adjusted for antihypertensive drugs and lipid-lowering drugs. | | | | |
| Abbreviations: FBG: fasting blood glucose; CVD: cardiovascular disease; T2D: type 2 diabetes; CKD: chronic kidney disease; IR: incidence rate. | | | | |

**Supplemental Table 8. Association Between Impaired FBG Progression and Risk of CVD-T2D, CKD-T2D, and CVD-CKD Multimorbidity Stratified by Follow-up Time Periods**

| **A.** | | | | |
| --- | --- | --- | --- | --- |
| **CVD-T2D** | | | | |
|  | **Sustained Normal FBG** | **Impaired FBG Progression** | **Impaired FBG Recovery** | **Persistent Impaired FBG** |
| **Follow-up Time from 0 to 5 years** | | | | |
| Events/Total | 22/27,105 | 29/7,832 | 7/4,157 | 19/3,979 |
| IR, per 1,000 person-years | 0.16 | 0.75 | 0.34 | 0.97 |
| Model 1 | 1.00 (reference) | 4.58 (2.63-7.97) | 2.08 (0.89-4.87) | 5.90 (3.20-10.91) |
| Model 2 | 1.00 (reference) | 3.88 (2.22-6.77) | 2.08 (0.89-4.89) | 5.31 (2.86-9.84) |
| Model 3 | 1.00 (reference) | 3.61 (2.06-6.32) | 2.00 (0.85-4.68) | 4.78 (2.57-8.90) |
| Model 4 | 1.00 (reference) | 3.57 (2.04-6.26) | 1.97 (0.84-4.62) | 4.70 (2.52-8.75) |
| **Follow-up Time > 5 years** | | | | |
| Events/Total | 142/26,397 | 135/7,528 | 47/4,025 | 128/3,789 |
| IR, per 1,000 person-years | 0.91 | 3.07 | 2.00 | 5.76 |
| Model 1 | 1.00 (reference) | 3.50 (2.76-4.44) | 2.19 (1.58-3.06) | 6.97 (5.47-8.86) |
| Model 2 | 1.00 (reference) | 3.18 (2.51-4.04) | 2.16 (1.55-3.01) | 6.35 (4.98-8.09) |
| Model 3 | 1.00 (reference) | 2.91 (2.29-3.69) | 2.07 (1.49-2.89) | 5.64 (4.42-7.20) |
| Model 4 | 1.00 (reference) | 2.88 (2.26-3.66) | 2.06 (1.48-2.88) | 5.56 (4.35-7.11) |

| **CKD-T2D** | | | | |
| --- | --- | --- | --- | --- |
|  | **Sustained Normal FBG** | **Impaired FBG Progression** | **Impaired FBG Recovery** | **Persistent Impaired FBG** |
| **Follow-up Time from 0 to 5 years** | | | | |
| Events/Total | 75/27,105 | 62/7,832 | 20/4,157 | 70/3,979 |
| IR, per 1,000 person-years | 0.56 | 1.61 | 0.97 | 3.57 |
| Model 1 | 1.00 (reference) | 2.88 (2.05-4.03) | 1.74 (1.06-2.85) | 6.41 (4.63-8.88) |
| Model 2 | 1.00 (reference) | 2.67 (1.90-2.95) | 1.80 (1.10-2.95) | 6.24 (4.49-8.68) |
| Model 3 | 1.00 (reference) | 2.38 (1.69-3.35) | 1.72 (1.05-2.82) | 5.47 (3.93-7.62) |
| Model 4 | 1.00 (reference) | 2.37 (1.68-3.32) | 1.70 (1.04-2.79) | 5.40 (3.87-7.52) |
| **Follow-up Time > 5 years** | | | | |
| Events/Total | 283/26,397 | 193/7,528 | 80/4,025 | 162/3,789 |
| IR, per 1,000 person-years | 1.83 | 4.42 | 3.44 | 7.43 |
| Model 1 | 1.00 (reference) | 2.27 (1.89-2.73) | 1.85 (1.45-2.37) | 3.89 (3.20-4.72) |
| Model 2 | 1.00 (reference) | 2.12 (1.76-2.56) | 1.85 (1.45-2.38) | 3.73 (3.07-4.54) |
| Model 3 | 1.00 (reference) | 2.01 (1.67-2.42) | 1.80 (1.40-2.31) | 3.41 (2.80-4.17) |
| Model 4 | 1.00 (reference) | 2.01 (1.67-2.42) | 1.79 (1.40-2.30) | 3.40 (2.78-4.15) |

| **CVD-CKD** | | | | |
| --- | --- | --- | --- | --- |
|  | **Sustained Normal FBG** | **Impaired FBG Progression** | **Impaired FBG Recovery** | **Persistent Impaired FBG** |
| **Follow-up Time from 0 to 5 years** | | | | |
| Events/Total | 58/27,105 | 32/7,832 | 9/4,157 | 11/3,979 |
| IR, per 1,000 person-years | 0.43 | 0.83 | 0.44 | 0.56 |
| Model 1 | 1.00 (reference) | 1.92 (1.24-2.95) | 1.01 (0.50-2.04) | 1.29 (0.68-2.46) |
| Model 2 | 1.00 (reference) | 1.59 (1.03-2.45) | 1.02 (0.51-2.06) | 1.15 (0.61-2.21) |
| Model 3 | 1.00 (reference) | 1.42 (0.92-2.20) | 0.98 (0.48-1.98) | 1.01 (0.53-1.93) |
| Model 4 | 1.00 (reference) | 1.39 (0.90-2.16) | 0.94 (0.46-1.90) | 0.96 (0.50-1.84) |
| **Follow-up Time > 5 years** | | | | |
| Events/Total | 237/26,397 | 108/7,528 | 49/4,025 | 63/3,789 |
| IR, per 1,000 person-years | 1.53 | 2.45 | 2.09 | 2.82 |
| Model 1 | 1.00 (reference) | 1.67 (1.33-2.10) | 1.33 (0.98-1.81) | 1.93 (1.46-2.55) |
| Model 2 | 1.00 (reference) | 1.44 (1.14-1.81) | 1.36 (1.00-1.86) | 1.76 (1.33-2.32) |
| Model 3 | 1.00 (reference) | 1.31 (1.04-1.65) | 1.32 (0.97-1.80) | 1.57 (1.18-2.08) |
| Model 4 | 1.00 (reference) | 1.29 (1.03-1.63) | 1.31 (0.96-1.79) | 1.53 (1.16-2.03) |

| **B.** | | | | |
| --- | --- | --- | --- | --- |
| **CVD-T2D** | | | | |
|  | **Sustained Normal FBG** | **Impaired FBG Progression** | **Impaired FBG Recovery** | **Persistent Impaired FBG** |
| **Follow-up Time from 0 to 5 years** | | | | |
| Events/Total | 22/27,105 | 29/7,832 | 7/4,157 | 19/3,979 |
| IR, per 1,000 person-years | 0.16 | 0.75 | 0.34 | 0.97 |
| Model 1 | 0.48 (0.21-1.13) | 2.20 (0.96-5.03) | 1.00 (reference) | 2.84 (1.19-6.75) |
| Model 2 | 0.48 (0.21-1.13) | 1.86 (0.81-4.26) | 1.00 (reference) | 2.55 (1.07-6.06) |
| Model 3 | 0.50 (0.21-1.17) | 1.81 (0.79-4.14) | 1.00 (reference) | 2.39 (1.00-5.71) |
| Model 4 | 0.50 (0.21-1.19) | 1.81 (0.79-4.16) | 1.00 (reference) | 2.39 (1.00-5.70) |
| **Follow-up Time > 5 years** | | | | |
| Events/Total | 142/26,397 | 135/7,528 | 47/4,025 | 128/3,789 |
| IR, per 1,000 person-years | 0.91 | 3.07 | 2.00 | 5.76 |
| Model 1 | 0.46 (0.33-0.63) | 1.59 (1.14-2.22) | 1.00 (reference) | 3.17 (2.27-4.44) |
| Model 2 | 0.46 (0.33-0.65) | 1.47 (1.05-2.06) | 1.00 (reference) | 2.94 (2.10-4.11) |
| Model 3 | 0.48 (0.35-0.67) | 1.40 (1.00-1.96) | 1.00 (reference) | 2.72 (1.94-3.81) |
| Model 4 | 0.48 (0.35-0.68) | 1.40 (1.00-1.95) | 1.00 (reference) | 2.70 (1.93-3.78) |

| **CKD-T2D** | | | | |
| --- | --- | --- | --- | --- |
|  | **Sustained Normal FBG** | **Impaired FBG Progression** | **Impaired FBG Recovery** | **Persistent Impaired FBG** |
| **Follow-up Time from 0 to 5 years** | | | | |
| Events/Total | 75/27,105 | 62/7,832 | 20/4,157 | 70/3,979 |
| IR, per 1,000 person-years | 0.56 | 1.61 | 0.97 | 3.57 |
| Model 1 | 0.57 (0.35-0.94) | 1.65 (1.00-2.73) | 1.00 (reference) | 3.68 (2.24-6.05) |
| Model 2 | 0.56 (0.34-0.91) | 1.48 (0.89-2.46) | 1.00 (reference) | 3.47 (2.11-5.71) |
| Model 3 | 0.58 (0.35-0.95) | 1.38 (0.83-2.30) | 1.00 (reference) | 3.18 (1.93-5.23) |
| Model 4 | 0.59 (0.36-0.96) | 1.39 (0.84-2.31) | 1.00 (reference) | 3.17 (1.93-5.23) |
| **Follow-up Time > 5 years** | | | | |
| Events/Total | 283/26,397 | 193/7,528 | 80/4,025 | 162/3,789 |
| IR, per 1,000 person-years | 1.83 | 4.42 | 3.44 | 7.43 |
| Model 1 | 0.54 (0.42-0.69) | 1.22 (0.94-1.59) | 1.00 (reference) | 2.10 (1.60-2.74) |
| Model 2 | 0.54 (0.42-0.69) | 1.14 (0.88-1.49) | 1.00 (reference) | 2.01 (1.54-2.63) |
| Model 3 | 0.56 (0.43-0.71) | 1.12 (0.86-1.45) | 1.00 (reference) | 1.90 (1.45-2.49) |
| Model 4 | 0.56 (0.43-0.71) | 1.12 (0.86-1.46) | 1.00 (reference) | 1.89 (1.44-2.49) |

| **CVD-CKD** | | | | |
| --- | --- | --- | --- | --- |
|  | **Sustained Normal FBG** | **Impaired FBG Progression** | **Impaired FBG Recovery** | **Persistent Impaired FBG** |
| **Follow-up Time from 0 to 5 years** | | | | |
| Events/Total | 58/27,105 | 32/7,832 | 9/4,157 | 11/3,979 |
| IR, per 1,000 person-years | 0.43 | 0.83 | 0.44 | 0.56 |
| Model 1 | 0.99 (0.49-1.99) | 1.89 (0.90-3.96) | 1.00 (reference) | 1.28 (0.53-3.08) |
| Model 2 | 0.98 (0.48-1.98) | 1.55 (0.74-3.26) | 1.00 (reference) | 1.13 (0.47-2.73) |
| Model 3 | 1.02 (0.51-2.06) | 1.45 (0.69-3.05) | 1.00 (reference) | 1.03 (0.43-2.49) |
| Model 4 | 1.07 (0.53-2.15) | 1.48 (0.70-3.12) | 1.00 (reference) | 1.02 (0.42-2.47) |
| **Follow-up Time > 5 years** | | | | |
| Events/Total | 237/26,397 | 108/7,528 | 49/4,025 | 63/3,789 |
| IR, per 1,000 person-years | 1.53 | 2.45 | 2.09 | 2.82 |
| Model 1 | 0.75 (0.55-1.03) | 1.26 (0.89-1.76) | 1.00 (reference) | 1.45 (1.00-2.11) |
| Model 2 | 0.73 (0.54-1.00) | 1.05 (0.75-1.48) | 1.00 (reference) | 1.29 (0.89-1.87) |
| Model 3 | 0.76 (0.56-1.03) | 0.99 (0.71-1.40) | 1.00 (reference) | 1.19 (0.81-1.73) |
| Model 4 | 0.76 (0.56-1.04) | 0.99 (0.70-1.39) | 1.00 (reference) | 1.17 (0.80-1.70) |

| (A) The sustained normal FBG group is used as the reference. (B) The impaired FBG recovery group is used as the reference. |
| --- |
| Model 1 was an unadjusted crude model. |
| Model 2 was further adjusted for age, sex, education level, drink status, smoking status and physical activity. |
| Model 3 was further adjusted for body mass index, high-density lipoprotein cholesterol, low-density lipoprotein cholesterol and high- sensitivity C-reactive protein. |
| Model 4 was further adjusted for antihypertensive drugs and lipid-lowering drugs. |
| Abbreviations: FBG: fasting blood glucose; CVD: cardiovascular disease; T2D: type 2 diabetes; CKD: chronic kidney disease; IR: incidence rate. |

**Supplemental Table 9. Association Between Impaired FBG Progression and Risk of Cardiovascular Disease, Chronic Kidney Disease and Type 2 Diabetes**

| **A.** | | | | |
| --- | --- | --- | --- | --- |
| **Cardiovascular Disease** | | | | |
|  | **Sustained Normal FBG** | **Impaired FBG Progression** | **Impaired FBG Recovery** | **Persistent Impaired FBG** |
| Events/Total | 1,883/27,098 | 705/7,832 | 337/4,157 | 378/3,979 |
| IR, per 1,000 person-years | 6.65 | 8.77 | 7.84 | 9.24 |
| Model 1 | 1.00 (reference) | 1.35 (1.24-1.47) | 1.18 (1.05-1.32) | 1.43 (1.28-1.60) |
| Model 2 | 1.00 (reference) | 1.15 (1.05-1.25) | 1.17 (1.04-1.31) | 1.26 (1.13-1.41) |
| Model 3 | 1.00 (reference) | 1.08 (0.99-1.18) | 1.14 (1.01-1.28) | 1.16 (1.04-1.30) |
| Model 4 | 1.00 (reference) | 1.07 (0.98-1.17) | 1.12 (1.00-1.26) | 1.14 (1.02-1.28) |
|  |  |  |  |  |
| **Chronic Kidney Disease** | | | | |
|  | **Sustained Normal FBG** | **Impaired FBG Progression** | **Impaired FBG Recovery** | **Persistent Impaired FBG** |
| Events/Total | 3,691/27,098 | 1,130/7,832 | 662/4,157 | 616/3,979 |
| IR, per 1,000 person-years | 13.62 | 14.58 | 16.26 | 15.76 |
| Model 1 | 1.00 (reference) | 1.08 (1.01-1.15) | 1.18 (1.09-1.28) | 1.16 (1.06-1.26) |
| Model 2 | 1.00 (reference) | 1.05 (0.98-1.12) | 1.19 (1.09-1.29) | 1.14 (1.05-1.24) |
| Model 3 | 1.00 (reference) | 1.04 (0.97-1.11) | 1.19 (1.09-1.29) | 1.13 (1.03-1.23) |
| Model 4 | 1.00 (reference) | 1.03 (0.97-1.11) | 1.18 (1.09-1.28) | 1.12 (1.02-1.22) |

| **Type 2 Diabetes** | | | | |
| --- | --- | --- | --- | --- |
|  | **Sustained Normal FBG** | **Impaired FBG Progression** | **Impaired FBG Recovery** | **Persistent Impaired FBG** |
| Events/Total | 1,709/27,098 | 1,483/7,832 | 578/4,157 | 1,305/3,979 |
| IR, per 1,000 person-years | 5.89 | 17.79 | 13.07 | 30.75 |
| Model 1 | 1.00 (reference) | 3.14 (2.93-3.37) | 2.28 (2.08-2.51) | 6.02 (5.60-6.48) |
| Model 2 | 1.00 (reference) | 3.06 (2.85-3.28) | 2.28 (2.07-2.50) | 5.89 (5.47-6.34) |
| Model 3 | 1.00 (reference) | 3.06 (2.85-3.28) | 2.28 (2.09-2.50) | 5.89 (5.47-6.34) |
| Model 4 | 1.00 (reference) | 2.89 (2.69-3.10) | 2.20 (2.00-2.42) | 5.50 (5.11-5.93) |

| **B.** | | | | |
| --- | --- | --- | --- | --- |
| **Cardiovascular Disease** | | | | |
|  | **Sustained Normal FBG** | **Impaired FBG Progression** | **Impaired FBG Recovery** | **Persistent Impaired FBG** |
| Events/Total | 1,883/27,098 | 705/7,832 | 337/4,157 | 378/3,979 |
| IR, per 1,000 person-years | 6.65 | 8.77 | 7.84 | 9.24 |
| Model 1 | 0.85 (0.76-0.95) | 1.15 (1.01-1.31) | 1.00 (reference) | 1.21 (1.04-1.41) |
| Model 2 | 0.86 (0.76-0.96) | 0.98 (0.86-1.12) | 1.00 (reference) | 1.08 (0.93-1.12) |
| Model 3 | 0.88 (0.78-0.99) | 0.95 (0.83-1.08) | 1.00 (reference) | 1.02 (0.88-1.18) |
| Model 4 | 0.89 (0.79-1.00) | 0.95 (0.84-1.09) | 1.00 (reference) | 1.02 (0.88-1.18) |
|  |  |  |  |  |
| **Chronic Kidney Disease** | | | | |
|  | **Sustained Normal FBG** | **Impaired FBG Progression** | **Impaired FBG Recovery** | **Persistent Impaired FBG** |
| Events/Total | 3,691/27,098 | 1,130/7,832 | 662/4,157 | 616/3,979 |
| IR, per 1,000 person-years | 13.62 | 14.58 | 16.26 | 15.76 |
| Model 1 | 0.85 (0.78-0.92) | 0.91 (0.83-1.00) | 1.00 (reference) | 0.98 (0.88-1.09) |
| Model 2 | 0.84 (0.77-0.91) | 0.88 (0.80-0.97) | 1.00 (reference) | 0.95 (0.86-1.07) |
| Model 3 | 0.84 (0.78-0.92) | 0.88 (0.80-0.96) | 1.00 (reference) | 0.95 (0.85-1.06) |
| Model 4 | 0.85 (0.78-0.92) | 0.88 (0.80-0.96) | 1.00 (reference) | 0.95 (0.85-1.06) |

| **Type 2 Diabetes** | | | | |
| --- | --- | --- | --- | --- |
|  | **Sustained Normal FBG** | **Impaired FBG Progression** | **Impaired FBG Recovery** | **Persistent Impaired FBG** |
| Events/Total | 1,709/27,098 | 1,483/7,832 | 578/4,157 | 1,305/3,979 |
| IR, per 1,000 person-years | 5.89 | 17.79 | 13.07 | 30.75 |
| Model 1 | 0.44 (0.40-0.48) | 1.38 (1.25-1.52) | 1.00 (reference) | 2.64 (2.39-2.91) |
| Model 2 | 0.44 (0.40-0.48) | 1.34 (1.22-1.48) | 1.00 (reference) | 2.59 (2.34-2.85) |
| Model 3 | 0.44 (0.40-0.48) | 1.34 (1.22-1.48) | 1.00 (reference) | 2.59 (2.34-2.85) |
| Model 4 | 0.45 (0.41-0.50) | 1.31 (1.19-1.44) | 1.00 (reference) | 2.50 (2.27-2.76) |
| (A) The sustained normal FBG group is used as the reference. (B) The impaired FBG recovery group is used as the reference. | | | | |
| Model 1 was an unadjusted crude model. | |  |  |  |
| Model 2 was further adjusted for age, sex, education level, drink status, smoking status and physical activity. | | | | |
| Model 3 was further adjusted for body mass index, high-density lipoprotein cholesterol, low-density lipoprotein cholesterol and high- sensitivity C-reactive protein. | | | | |
| Model 4 was further adjusted for antihypertensive drugs and lipid-lowering drugs. | | | | |
| Abbreviations: FBG: fasting blood glucose; IR: incidence rate. | | | | |

**Supplemental Table 10. Association Between Impaired FBG Progression and Risk of Cardiovascular Disease, Chronic Kidney Disease and Type 2 Diabetes Stratified by Follow-up Time Periods**

| **A.** | | | | |
| --- | --- | --- | --- | --- |
| **Cardiovascular Disease** | | | | |
|  | **Sustained Normal FBG** | **Impaired FBG Progression** | **Impaired FBG Recovery** | **Persistent Impaired FBG** |
| **Follow-up Time from 0 to 5 years** | | | | |
| Events/Total | 567/27,105 | 253/7,832 | 95/4,157 | 95/3,979 |
| IR, per 1,000 person-years | 4.27 | 6.63 | 4.67 | 5.40 |
| Model 1 | 1.00 (reference) | 1.55 (1.34-1.80) | 1.09 (0.88-1.36) | 1.27 (1.03-1.56) |
| Model 2 | 1.00 (reference) | 1.29 (1.11-1.50) | 1.10 (0.89-1.37) | 1.13 (0.92-1.39) |
| Model 3 | 1.00 (reference) | 1.20 (1.03-1.40) | 1.07 (0.86-1.34) | 1.04 (0.84-1.28) |
| Model 4 | 1.00 (reference) | 1.19 (1.03-1.39) | 1.05 (0.85-1.31) | 1.01 (0.82-1.25) |
| **Follow-up Time > 5 years** | | | | |
| Events/Total | 1,316/26,397 | 452/7,528 | 242/4,025 | 273/3,789 |
| IR, per 1,000 person-years | 8.76 | 10.70 | 10.70 | 12.70 |
| Model 1 | 1.00 (reference) | 1.26 (1.13-1.40) | 1.21 (1.06-1.39) | 1.51 (1.32-1.72) |
| Model 2 | 1.00 (reference) | 1.08 (0.97-1.20) | 1.19 (1.04-1.37) | 1.32 (1.16-1.51) |
| Model 3 | 1.00 (reference) | 1.02 (0.91-1.13) | 1.17 (1.02-1.34) | 1.22 (1.07-1.39) |
| Model 4 | 1.00 (reference) | 1.01 (0.91-1.13) | 1.15 (1.01-1.32) | 1.20 (1.06-1.37) |
|  |  |  |  |  |
| **Chronic Kidney Disease** | | | | |
|  | **Sustained Normal FBG** | **Impaired FBG Progression** | **Impaired FBG Recovery** | **Persistent Impaired FBG** |
| **Follow-up Time from 0 to 5 years** | | | | |
| Events/Total | 1,782/27,105 | 547/7,832 | 338/4,157 | 323/3,979 |
| IR, per 1,000 person-years | 13.60 | 14.50 | 17.00 | 16.90 |
| Model 1 | 1.00 (reference) | 1.06 (0.97-1.17) | 1.25 (1.11-1.40) | 1.24 (1.10-1.40) |
| Model 2 | 1.00 (reference) | 1.03 (0.94-1.14) | 1.27 (1.13-1.43) | 1.23 (1.09-1.38) |
| Model 3 | 1.00 (reference) | 1.02 (0.93-1.13) | 1.26 (1.08-1.37) | 1.22 (1.08-1.37) |
| Model 4 | 1.00 (reference) | 1.02 (0.93-1.13) | 1.26 (1.12-1.41) | 1.21 (1.07-1.36) |
| **Follow-up Time > 5 years** | | | | |
| Events/Total | 1,909/26,397 | 583/7,528 | 324/4,025 | 293/3,789 |
| IR, per 1,000 person-years | 13.60 | 14.60 | 15.60 | 14.70 |
| Model 1 | 1.00 (reference) | 1.08 (0.99-1.19) | 1.12 (0.99-1.25) | 1.07 (0.95-1.21) |
| Model 2 | 1.00 (reference) | 1.06 (0.97-1.17) | 1.11 (0.99-1.25) | 1.06 (0.93-1.20) |
| Model 3 | 1.00 (reference) | 1.05 (0.96-1.15) | 1.11 (0.99-1.25) | 1.04 (0.92-1.18) |
| Model 4 | 1.00 (reference) | 1.04 (0.95-1.15) | 1.11 (0.98-1.24) | 1.03 (0.91-1.17) |

| **Type 2 Diabetes** | | | | |
| --- | --- | --- | --- | --- |
|  | **Sustained Normal FBG** | **Impaired FBG Progression** | **Impaired FBG Recovery** | **Persistent Impaired FBG** |
| **Follow-up Time from 0 to 5 years** | | | | |
| Events/Total | 571/27,105 | 712/7,832 | 244/4,157 | 696/3,979 |
| IR, per 1,000 person-years | 4.29 | 19.10 | 12.10 | 38.10 |
| Model 1 | 1.00 (reference) | 4.50 (4.03-5.02) | 2.84 (2.44-3.30) | 9.10 (8.15-10.17) |
| Model 2 | 1.00 (reference) | 4.28 (3.84-4.79) | 2.84 (2.45-3.31) | 8.80 (7.87-9.84) |
| Model 3 | 1.00 (reference) | 4.02 (3.60-4.49) | 2.76 (2.37-3.21) | 8.18 (7.31-9.15) |
| Model 4 | 1.00 (reference) | 4.01 (3.59-4.49) | 2.75 (2.37-3.20) | 8.15 (7.28-9.13) |
| **Follow-up Time > 5 years** | | | | |
| Events/Total | 1,138/26,397 | 771/7,528 | 334/4,025 | 609/3,789 |
| IR, per 1,000 person-years | 7.59 | 20.00 | 15.60 | 35.90 |
| Model 1 | 1.00 (reference) | 2.45 (2.23-2.69) | 2.00 (1.77-2.26) | 4.38 (3.96-4.83) |
| Model 2 | 1.00 (reference) | 2.42 (2.21-2.66) | 1.99 (1.76-2.25) | 4.33 (3.91-4.79) |
| Model 3 | 1.00 (reference) | 2.31 (2.10-2.53) | 1.94 (1.71-2.19) | 4.11 (3.71-4.55) |
| Model 4 | 1.00 (reference) | 2.30 (2.09-2.52) | 1.93 (1.70-2.18) | 4.09 (3.69-4.52) |

| **B.** | | | | |
| --- | --- | --- | --- | --- |
| **Cardiovascular Disease** | | | | |
|  | **Sustained Normal FBG** | **Impaired FBG Progression** | **Impaired FBG Recovery** | **Persistent Impaired FBG** |
| **Follow-up Time from 0 to 5 years** |  |  |  |  |
| Events/Total | 1,883/27,098 | 705/7,832 | 337/4,157 | 378/3,979 |
| IR, per 1,000 person-years | 6.65 | 8.77 | 7.84 | 9.24 |
| Model 1 | 0.85 (0.76-0.95) | 1.15 (1.01-1.31) | 1.00 (reference) | 1.21 (1.04-1.41) |
| Model 2 | 0.86 (0.76-0.96) | 0.98 (0.86-1.12) | 1.00 (reference) | 1.08 (0.93-1.12) |
| Model 3 | 0.88 (0.78-0.99) | 0.95 (0.83-1.08) | 1.00 (reference) | 1.02 (0.88-1.18) |
| Model 4 | 0.89 (0.79-1.00) | 0.95 (0.84-1.09) | 1.00 (reference) | 1.02 (0.88-1.18) |
| **Follow-up Time > 5 years** | | | | |
| Events/Total | 1,316/26,397 | 452/7,528 | 242/4,025 | 273/3,789 |
| IR, per 1,000 person-years | 8.76 | 10.70 | 10.70 | 12.70 |
| Model 1 | 0.85 (0.78-0.92) | 0.91 (0.83-1.00) | 1.00 (reference) | 0.98 (0.88-1.09) |
| Model 2 | 0.84 (0.77-0.91) | 0.88 (0.80-0.97) | 1.00 (reference) | 0.95 (0.86-1.07) |
| Model 3 | 0.84 (0.78-0.92) | 0.88 (0.80-0.96) | 1.00 (reference) | 0.95 (0.85-1.06) |
| Model 4 | 0.85 (0.78-0.92) | 0.88 (0.80-0.96) | 1.00 (reference) | 0.95 (0.85-1.06) |

| **Chronic Kidney Disease** | | | | |
| --- | --- | --- | --- | --- |
|  | **Sustained Normal FBG** | **Impaired FBG Progression** | **Impaired FBG Recovery** | **Persistent Impaired FBG** |
| **Follow-up Time from 0 to 5 years** | | | | |
| Events/Total | 1,782/27,105 | 547/7,832 | 338/4,157 | 323/3,979 |
| IR, per 1,000 person-years | 13.60 | 14.50 | 17.00 | 16.90 |
| Model 1 | 0.80 (0.71-0.90) | 0.85 (0.74-0.98) | 1.00 (reference) | 0.99 (0.85-1.16) |
| Model 2 | 0.79 (0.70-0.88) | 0.81 (0.71-0.93) | 1.00 (reference) | 0.96 (0.83-1.12) |
| Model 3 | 0.79 (0.70-0.89) | 0.81 (0.71-0.93) | 1.00 (reference) | 0.96 (0.82-1.12) |
| Model 4 | 0.79 (0.71-0.89) | 0.81 (0.71-0.93) | 1.00 (reference) | 0.96 (0.82-1.12) |
| **Follow-up Time > 5 years** | | | | |
| Events/Total | 1,909/26,397 | 583/7,528 | 324/4,025 | 293/3,789 |
| IR, per 1,000 person-years | 13.60 | 14.60 | 15.60 | 14.70 |
| Model 1 | 0.90 (0.80-1.01) | 0.97 (0.85-1.11) | 1.00 (reference) | 0.96 (0.82-1.13) |
| Model 2 | 0.90 (0.80–1.01) | 0.96 (0.83–1.10) | 1.00 (reference) | 0.95 (0.81–1.11) |
| Model 3 | 0.90 (0.80–1.01) | 0.95 (0.83–1.08) | 1.00 (reference) | 0.94 (0.80–1.10) |
| Model 4 | 0.90 (0.80–1.02) | 0.95 (0.83–1.09) | 1.00 (reference) | 0.93 (0.79–1.09) |

| **Type 2 Diabetes** | | | | | | | |
| --- | --- | --- | --- | --- | --- | --- | --- |
|  | **Sustained Normal FBG** | | **Impaired FBG Progression** | | **Impaired FBG Recovery** | | **Persistent Impaired FBG** |
| **Follow-up Time from 0 to 5 years** | | | | | | | |
| Events/Total | 571/27,105 | | 712/7,832 | | 244/4,157 | | 696/3,979 |
| IR, per 1,000 person-years | 4.29 | | 19.10 | | 12.10 | | 38.10 |
| Model 1 | 0.35 (0.30–0.41) | | 1.58 (1.37–1.83) | | 1.00 (reference) | | 3.20 (2.77–3.71) |
| Model 2 | 0.35 (0.30–0.41) | | 1.51 (1.30–1.74) | | 1.00 (reference) | | 3.09 (2.67–3.58) |
| Model 3 | 0.36 (0.31–0.42) | | 1.46 (1.26–1.69) | | 1.00 (reference) | | 2.96 (2.56–3.43) |
| Model 4 | 0.36 (0.31–0.42) | | 1.46 (1.26–1.69) | | 1.00 (reference) | | 2.96 (2.56–3.43) |
| **Follow-up Time > 5 years** | | | | | | | |
| Events/Total | 1,138/26,397 | | 771/7,528 | | 334/4,025 | | 609/3,789 |
| IR, per 1,000 person-years | 7.59 | | 20.00 | | 15.60 | | 35.90 |
| Model 1 | 0.50 (0.44–0.57) | | 1.22 (1.08–1.39) | | 1.00 (reference) | | 2.19 (1.91–2.50) |
| Model 2 | 0.50 (0.44–0.57) | | 1.22 (1.07–1.38) | | 1.00 (reference) | | 2.17 (1.90–2.49) |
| Model 3 | 0.52 (0.46–0.58) | | 1.19 (1.05–1.36) | | 1.00 (reference) | | 2.12 (1.85–2.43) |
| Model 4 | 0.52 (0.46–0.59) | | 1.19 (1.05–1.36) | | 1.00 (reference) | | 2.12 (1.85–2.43) |
| (A) The sustained normal FBG group is used as the reference. (B) The impaired FBG recovery group is used as the reference. | | | | | | | |
| Model 1 was an unadjusted crude model. | |  | |  | |  | |
| Model 2 was further adjusted for age, sex, education level, drink status, smoking status and physical activity. | | | | | | | |
| Model 3 was further adjusted for body mass index, high-density lipoprotein cholesterol, low-density lipoprotein cholesterol and high- sensitivity C-reactive protein. | | | | | | | |
| Model 4 was further adjusted for antihypertensive drugs and lipid-lowering drugs. | | | | | | | |
| Abbreviations: FBG: fasting blood glucose; IR: incidence rate. | | | | | | | |

**Supplemental Table 11. Stratified Analysis**

| **A.** | |  |  |  |  |
| --- | --- | --- | --- | --- | --- |
|  | **Sustained Normal FBG** | **Impaired FBG Progression** | **Impaired FBG Recovery** | **Persistent Impaired FBG** | ***P* for Interaction** |
| **Age** |  |  |  |  | 0.06 |
| <60y |  |  |  |  |  |
| Events/Total | 493/21,547 | 301/5,682 | 146/3,468 | 284/3,188 |  |
| IR, per 1,000 person-years | 2.11 | 4.89 | 3.91 | 8.28 |  |
| HRs (95% CIs) | 1.00 (reference) | 2.08 (1.81-2.42) | 1.74 (1.45-2.10) | 3.54 (3.05-4.11) |  |
|  |  |  |  |  |  |
| >60y |  |  |  |  |  |
| Events/Total | 244/5,558 | 178/2,150 | 52/689 | 97/791 |  |
| IR, per 1,000 person-years | 4.41 | 8.37 | 7.79 | 12.49 |  |
| HRs (95% CIs) | 1.00 (reference) | 1.82 (1.50-2.22) | 1.68 (1.24-2.26) | 2.67 (2.11-3.39) |  |
|  | | | | |  |
| **Sex** |  |  |  |  | 0.46 |
| Men |  |  |  |  |  |
| Events/Total | 607/19,932 | 392/6,364 | 174/3,486 | 337/3,470 |  |
| IR, per 1,000 person-years | 2.86 | 5.86 | 4.73 | 9.2 |  |
| HRs (95% CIs) | 1.00 (reference) | 1.85 (1.63-2.11) | 1.64 (1.39-1.94) | 3.02 (2.64-3.46) |  |
|  |  |  |  |  |  |
| Women |  |  |  |  |  |
| Events/Total | 130/7,173 | 87/1,468 | 24/671 | 44/509 |  |
| IR, per 1,000 person-years | 1.68 | 5.5 | 3.34 | 8.07 |  |
| HRs (95% CIs) | 1.00 (reference) | 2.37 (1.79-3.14) | 2.05 (1.33-3.18) | 3.71 (2.62-5.27) |  |
|  |  |  |  |  |  |
| **BMI** |  |  |  |  | 0.15 |
| >=28 kg/m² |  |  |  |  |  |
| Events/Total | 97/3,850 | 104/1,546 | 27/680 | 65/841 |  |
| IR, per 1,000 person-years | 2.35 | 6.30 | 3.72 | 7.32 |  |
| HRs (95% CIs) | 1.00 (reference) | 2.61 (2.11-3.23) | 1.72 (1.25-2.37) | 3.22 (2.53-4.10) |  |
| <28 kg/m² |  |  |  |  |  |
| Events/Total | 261/23,255 | 151/6,286 | 73/3,477 | 167/3,138 |  |
| IR, per 1,000 person-years | 1.05 | 2.26 | 1.98 | 4.99 |  |
| HRs (95% CIs) | 1.00 (reference) | 1.75 (1.52-2.02) | 1.72 (1.43-2.06) | 3.21 (2.77-3.72) |  |
|  |  |  |  |  |  |
| **WC** |  |  |  |  | 0.86 |
| men: >=90 cm; women: >=85cm |  |  |  |  |  |
| Events/Total | 68/2,221 | 60/699 | 14/226 | 26/263 |  |
| IR, per 1,000 person-years | 2.85 | 7.94 | 5.82 | 9.21 |  |
| HRs (95% CIs) | 1.00 (reference) | 2.04 (1.75-2.36) | 1.95 (1.60-2.38) | 3.02 (2.56-3.55) |  |
| men: <90 cm; women: <85cm |  |  |  |  |  |
| Events/Total | 669/24,884 | 419/7,133 | 184/3,931 | 355/3,716 |  |
| IR, per 1,000 person-years | 2.52 | 5.57 | 4.42 | 9.05 |  |
| HRs (95% CIs) | 1.00 (reference) | 1.62 (1.32-1.99) | 1.24 (0.94-1.64) | 2.93 (2.48-3.62) |  |
|  |  |  |  |  |  |
| **Antihypertensive Drugs** |  |  |  |  | <0.01 |
| Yes |  |  |  |  |  |
| Events/Total | 60/2,320 | 33/963 | 17/450 | 43/510 |  |
| IR, per 1,000 person-years | 2.50 | 3.28 | 3.70 | 8.01 |  |
| HRs (95% CIs) | 1.00 (reference) | 1.13 (0.86-1.49) | 1.36 (0.95-1.94) | 2.29 (1.74-3.03) |  |
| No |  |  |  |  |  |
| Events/Total | 298/24,785 | 222/6,869 | 83/3,707 | 189/3,469 |  |
| IR, per 1,000 person-years | 1.12 | 3.03 | 2.10 | 5.12 |  |
| HRs (95% CIs) | 1.00 (reference) | 2.19 (1.92-2.49) | 1.77 (1.48-2.11) | 3.34 (2.90-3.84) |  |
|  |  |  |  |  |  |
| **hs-CRP, mg/L** |  |  |  |  | 0.18 |
| ＞=3.00 mg/L |  |  |  |  |  |
| Events/Total | 130/6,168 | 95/1,977 | 32/965 | 78/943 |  |
| IR, per 1,000 person-years | 1.98 | 4.56 | 3.13 | 7.78 |  |
| HRs (95% CIs) | 1.00 (reference) | 1.92 (1.57-2.35) | 1.38 (1.03-1.86) | 3.09 (2.48-3.84) |  |
| ＜3.00 mg/L |  |  |  |  |  |
| Events/Total | 228/20,937 | 160/5,855 | 68/3,192 | 154/3,036 |  |
| IR, per 1,000 person-years | 1.02 | 2.56 | 2.00 | 4.77 |  |
| HRs (95% CIs) | 1.00 (reference) | 1.94 (1.68-2.24) | 1.84 (1.53-2.22) | 3.15 (2.70-3.67) |  |
|  | | | | | |

| **B.** | |  |  |  |  |
| --- | --- | --- | --- | --- | --- |
|  | **Sustained Normal FBG** | **Impaired FBG Progression** | **Impaired FBG Recovery** | **Persistent Impaired FBG** | ***P* for Interaction** |
| **Age** |  |  |  |  | 0.06 |
| <60y |  |  |  |  |  |
| Events/Total | 493/21,547 | 301/5,682 | 146/3,468 | 284/3,188 |  |
| IR, per 1,000 person-years | 2.11 | 4.89 | 3.91 | 8.28 |  |
| HRs (95% CIs) | 0.57 (0.48-0.69) | 1.20 (0.98-1.46) | 1.00 (reference) | 2.03 (1.66-2.48) |  |
|  |  |  |  |  |  |
| >60y |  |  |  |  |  |
| Events/Total | 244/5,558 | 178/2,150 | 52/689 | 97/791 |  |
| IR, per 1,000 person-years | 4.41 | 8.37 | 7.79 | 12.49 |  |
| HRs (95% CIs) | 0.60 (0.44-0.81) | 1.09 (0.80-1.48) | 1.00 (reference) | 1.59 (1.14-2.24) |  |
|  | | | | |  |
| **Sex** |  |  |  |  | 0.46 |
| Men |  |  |  |  |  |
| Events/Total | 607/19,932 | 392/6,364 | 174/3,486 | 337/3,470 |  |
| IR, per 1,000 person-years | 2.86 | 5.86 | 4.73 | 9.2 |  |
| HRs (95% CIs) | 0.61 (0.51-0.72) | 1.13 (0.94-1.35) | 1.00 (reference) | 1.84 (1.53-2.21) |  |
|  |  |  |  |  |  |
| Women |  |  |  |  |  |
| Events/Total | 130/7,173 | 87/1,468 | 24/671 | 44/509 |  |
| IR, per 1,000 person-years | 1.68 | 5.5 | 3.34 | 8.07 |  |
| HRs (95% CIs) | 0.49 (0.31-0.75) | 1.15 (0.73-1.82) | 1.00 (reference) | 1.81 (1.09-2.99) |  |
|  |  |  |  |  |  |
| **BMI** |  |  |  |  | 0.15 |
| >=28 kg/m² |  |  |  |  |  |
| Events/Total | 97/3,850 | 104/1,546 | 27/680 | 65/841 |  |
| IR, per 1,000 person-years | 2.35 | 6.30 | 3.72 | 7.32 |  |
| HRs (95% CIs) | 0.58 (0.42-0.80) | 1.51 (1.10-2.08) | 1.00 (reference) | 1.87 (1.33-2.61) |  |
| <28 kg/m² |  |  |  |  |  |
| Events/Total | 261/23,255 | 151/6,286 | 73/3,477 | 167/3,138 |  |
| IR, per 1,000 person-years | 1.05 | 2.26 | 1.98 | 4.99 |  |
| HRs (95% CIs) | 0.58 (0.49-0.70) | 1.02 (0.84-1.24) | 1.00 (reference) | 1.87 (1.53-2.29) |  |
|  |  |  |  |  |  |
| **WC** |  |  |  |  | 0.86 |
| men: >=90 cm; women: >=85cm |  |  |  |  |  |
| Events/Total | 68/2,221 | 60/699 | 14/226 | 26/263 |  |
| IR, per 1,000 person-years | 2.85 | 7.94 | 5.82 | 9.21 |  |
| HRs (95% CIs) | 0.51 (0.42-0.63) | 1.04 (0.85-1.28) | 1.00 (reference) | 1.55 (1.25-1.92) |  |
| men: <90 cm; women: <85cm |  |  |  |  |  |
| Events/Total | 669/24,884 | 419/7,133 | 184/3,931 | 355/3,716 |  |
| IR, per 1,000 person-years | 2.52 | 5.57 | 4.42 | 9.05 |  |
| HRs (95% CIs) | 0.81 (0.61-1.07) | 1.31 (0.96-1.78) | 1.00 (reference) | 2.36 (1.74-3.22) |  |
|  |  |  |  |  |  |
| **Antihypertensive Drugs** |  |  |  |  | <0.01 |
| Yes |  |  |  |  |  |
| Events/Total | 60/2,320 | 33/963 | 17/450 | 43/510 |  |
| IR, per 1,000 person-years | 2.50 | 3.28 | 3.70 | 8.01 |  |
| HRs (95% CIs) | 0.74 (0.52-1.05) | 0.83 (0.56-1.23) | 1.00 (reference) | 1.69 (1.15-2.48) |  |
| No |  |  |  |  |  |
| Events/Total | 298/24,785 | 222/6,869 | 83/3,707 | 189/3,469 |  |
| IR, per 1,000 person-years | 1.12 | 3.03 | 2.10 | 5.12 |  |
| HRs (95% CIs) | 0.57 (0.47-0.67) | 1.24 (1.03-1.49) | 1.00 (reference) | 1.88 (1.55-2.29) |  |
|  |  |  |  |  |  |
| **hs-CRP, mg/L** |  |  |  |  | 0.18 |
| ＞=3.00 mg/L |  |  |  |  |  |
| Events/Total | 130/6,168 | 95/1,977 | 32/965 | 78/943 |  |
| IR, per 1,000 person-years | 1.98 | 4.56 | 3.13 | 7.78 |  |
| HRs (95% CIs) | 0.72 (0.54-0.97) | 1.39 (1.02-1.89) | 1.00 (reference) | 2.23 (1.62-3.08) |  |
| ＜3.00 mg/L |  |  |  |  |  |
| Events/Total | 228/20,937 | 160/5,855 | 68/3,192 | 154/3,036 |  |
| IR, per 1,000 person-years | 1.02 | 2.56 | 2.00 | 4.77 |  |
| HRs (95% CIs) | 0.54 (0.45-0.66) | 1.06 (0.87-1.29) | 1.00 (reference) | 1.71 (1.40-2.10) |  |
| (A) The sustained normal FBG group is used as the reference. (B) The impaired FBG recovery group is used as the reference. | | | | | |
| Adjusted for age, sex, education level, drink status, smoking status, physical activity, body mass index, high-density lipoprotein cholesterol, low-density lipoprotein cholesterol, high- sensitivity C-reactive protein, antihypertensive drugs and lipid-lowering drugs, except for the covariates used for stratification. | | | | | |
| Abbreviations: FBG: fasting blood glucose; IR: incidence rate; HR: hazard ratio; BMI: body index mass; WC: waist circumference; hs-CRP, high-sensitivity C-reactive protein. | | | | | |

**Supplemental Table 12. Sensitivity Analysis**

| **A.** | | | | |
| --- | --- | --- | --- | --- |
|  | **Sustained Normal FBG** | **Impaired FBG Progression** | **Impaired FBG Recovery** | **Persistent Impaired FBG** |
| **2-year Lagged Analysis** | | | | |
| Events/Total | 730/27,098 | 475/7,828 | 198/4,157 | 380/3,978 |
| IR, per 1,000 person-years | 2.52 | 5.74 | 4.50 | 9.03 |
| HRs (95% CI) | 1.00 (reference) | 1.94 (1.72-2.18) | 1.71 (1.46-2.00) | 3.04 (2.68-3.46) |
| **Excluded Participants with Cancer** | | | | |
| Events/Total | 697/25,785 | 467/7,421 | 188/3,944 | 356/3,752 |
| IR, per 1,000 person-years | 2.51 | 5.91 | 4.46 | 8.89 |
| HRs (95% CI) | 1.00 (reference) | 1.95 (1.73-2.20) | 1.68 (1.40-1.97) | 3.08 (2.17-3.63) |
| **Excluded Participants Receiving Antihypertensive Drug Treatment** | | | | |
| Events/Total | 583/24,785 | 402/6,869 | 159/3,707 | 302/3,469 |
| IR, per 1,000 person-years | 2.20 | 5.52 | 4.03 | 8.21 |
| HRs (95% CI) | 1.00 (reference) | 1.96 (1.73-2.22) | 1.69 (1.43-1.99) | 3.10 (2.72-3.54) |
| **Excluded Participants Receiving Lipid-lowering Drug Treatment** | | | | |
| Events/Total | 717/26,815 | 467/7,749 | 196/4,095 | 374/3,928 |
| IR, per 1,000 person-years | 2.51 | 5.70 | 4.52 | 9.00 |
| HRs (95% CI) | 1.00 (reference) | 1.93 (1.72-2.17) | 1.74 (1.48-2.03) | 3.14 (2.77-3.57) |
| **Groups of Impaired FBG Evolution based on the WHO Criteria** | | | | |
| Events/Total | 1,219/37,217 | 323/3,307 | 149/1,862 | 104/687 |
| IR, per 1,000 person-years | 3.07 | 9.31 | 7.61 | 14.63 |
| HRs (95% CI) | 1.00 (reference) | 1.93 (1.72-2.17) | 1.69 (1.44-1.98) | 3.10 (2.74-3.52) |
| **Groups of impaired FBG Evolution based on the Participants with Complete Data** | | | | |
| Events/Total | 642/23,684 | 435/6,954 | 176/3,655 | 345/3,600 |
| IR, per 1,000 person-years | 2.53 | 5.91 | 4.55 | 9.13 |
| HRs (95% CI) | 1.00 (reference) | 1.99 (1.76-2.26) | 1.72 (1.45-2.04) | 3.20 (2.81-3.66) |
| **Group of impaired FBG Evolution and Incident CKM Multimorbidity Using a Stricter Definition of CKD** | | | | |
| Events/Total | 230/27,105 | 196/7,832 | 61/4,157 | 162/3,979 |
| IR, per 1,000 person-years | 0.77 | 2.28 | 1.33 | 3.70 |
| HRs (95% CI) | 1.00 (reference) | 2.48 (2.05-3.00) | 1.61 (1.22-2.14) | 4.02 (3.28-4.93) |
| **Fine-Gray** | | | | |
| Events/Total | 4,447/27,105 | 1,741/7,832 | 802/4,157 | 927/3,979 |
| IR, per 1,000 person-years | 15.38 | 21.03 | 18.23 | 22.04 |
| HRs (95% CI) | 1 (reference) | 1.92 (1.71-2.16) | 1.66 (1.42-1.95) | 3.06 (2.69-3.47) |

| **B.** | | | | |
| --- | --- | --- | --- | --- |
|  | **Sustained Normal FBG** | **Impaired FBG Progression** | **Impaired FBG Recovery** | **Persistent Impaired FBG** |
| **2-year Lagged Analysis** | | | | |
| Events/Total | 730/27,098 | 475/7,828 | 198/4,157 | 380/3,978 |
| IR, per 1,000 person-years | 2.52 | 5.74 | 4.50 | 9.03 |
| HRs (95% CI) | 0.58 (0.50-0.68) | 1.13 (0.96-1.34) | 1.00 (reference) | 1.78 (1.49-2.12) |
| **Excluded Participants with Cancer** | | | | |
| Events/Total | 697/25,785 | 467/7,421 | 188/3,944 | 356/3,752 |
| IR, per 1,000 person-years | 2.51 | 5.91 | 4.46 | 8.89 |
| HRs (95% CI) | 0.60 (0.51-0.72) | 1.15 (0.97-1.38) | 1.00 (reference) | 1.82 (1.51-2.17) |
| **Excluded Participants Receiving Antihypertensive Drug Treatment** | | | | |
| Events/Total | 583/24,785 | 402/6,869 | 159/3,707 | 302/3,469 |
| IR, per 1,000 person-years | 2.20 | 5.52 | 4.03 | 8.21 |
| HRs (95% CI) | 0.59 (0.50-0.70) | 1.16 (0.98-1.39) | 1.00 (reference) | 1.84 (1.54-2.21) |
| **Excluded Participants Receiving Lipid-lowering Drug Treatment** | | | | |
| Events/Total | 717/26,815 | 467/7,749 | 196/4,095 | 374/3,928 |
| IR, per 1,000 person-years | 2.51 | 5.70 | 4.52 | 9.00 |
| HRs (95% CI) | 0.58 (0.49-0.68) | 1.11 (0.94-1.32) | 1.00 (reference) | 1.81 (1.52-2.15) |
| **Groups of Impaired FBG Evolution based on the WHO Criteria** | | | | |
| Events/Total | 1,219/37,217 | 323/3,307 | 149/1,862 | 104/687 |
| IR, per 1,000 person-years | 3.07 | 9.31 | 7.61 | 14.63 |
| HRs (95% CI) | 0.59 (0.51-0.69) | 1.14 (0.97-1.35) | 1.00 (reference) | 1.84 (1.55-2.18) |
| **Groups of impaired FBG Evolution based on the Participants with Complete Data** | | | | |
| Events/Total | 642/23,684 | 435/6,954 | 176/3,655 | 345/3,600 |
| IR, per 1,000 person-years | 2.53 | 5.91 | 4.55 | 9.13 |
| HRs (95% CI) | 0.58 (0.49-0.69) | 1.16 (0.97-1.38) | 1.00 (reference) | 1.86 (1.55-2.24) |
| **Group of impaired FBG Evolution and Incident CKM Multimorbidity Using a Stricter Definition of CKD** | | | | |
| Events/Total | 230/27,105 | 196/7,832 | 61/4,157 | 162/3,979 |
| IR, per 1,000 person-years | 0.77 | 2.28 | 1.33 | 3.70 |
| HRs (95% CI) | 0.62 (0.47-0.82) | 1.54 (1.15-2.05) | 1.00 (reference) | 2.49 (1.85-3.35) |
| **Fine-Gray** | | | | |
| Events/Total | 4,447/27,105 | 1,741/7,832 | 802/4,157 | 927/3,979 |
| IR, per 1,000 person-years | 15.38 | 21.03 | 18.23 | 22.04 |
| HRs (95% CI) | 0.60 (0.51-0.70) | 1.16 (0.98-1.37) | 1 (reference) | 1.84 (1.55-2.19) |
| (A) The sustained normal FBG group is used as the reference. (B) The impaired FBG recovery group is used as the reference. | | | | |
| Adjusted for age, sex, education level, drink status, smoking status, physical activity, body mass index, high-density lipoprotein cholesterol, low-density lipoprotein cholesterol, high- sensitivity C-reactive protein, antihypertensive drugs and lipid-lowering drugs. | | | | |
| Abbreviations: FBG: fasting blood glucose; IR: incidence rate; HR: hazard ratio; WHO: World Health Organization; CKM: Cardio-Kidney-Metabolic; CKD: chronic kidney disease. | | | | |

**Supplemental Figure 1. Study Flowchart**

Participants who took part in and completed health examinations in both 2006 and 2010. (N=68,150)

Participants included in the study follow-up up to December 31, 2021 (N=43,073); Median (IQR) follow-up, 11.00 (10.62-11.32) y

25,077 excluded

-Individuals missing fasting blood glucose data in either examination.

(N=935)

-Individuals diagnosed with cardiovascular disease in 2010 or earlier. (N=1,447)

-Individuals diagnosed with diabetes in 2010 or earlier. (N=11,387)

-Individuals diagnosed with chronic kidney disease in 2010 or earlier.

(N= 11,308)

Abbreviations: IQR, interquartile range.

**Supplemental Figure 2. Restricted Cubic Spline for Fasting Blood Glucose in 2010**

**
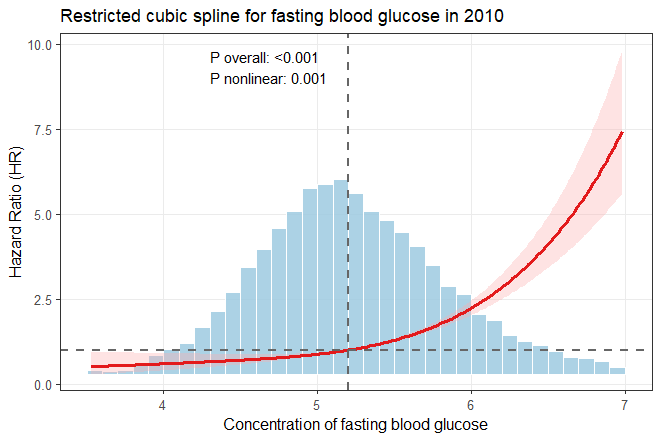
**

Restricted cubic splines were fitted with 3 knots placed at the 25th, 50th, and 75th percentiles.

The solid line represents the adjusted hazard ratio, and the shaded area represents the 95% confidence interval.

Models were adjusted for age, sex, education level, smoking status, drinking status, physical activity, body mass index, HDL-C, LDL-C, hs-CRP, antihypertensive drugs, and lipid-lowering drugs.

Abbreviations: HDL-C, high density lipoprotein cholesterol; LDL-C, low density lipoprotein cholesterol.

**Supplemental Figure 3. Multi-State Transition Patterns from Baseline to Cardio-Kidney-Metabolic Multimorbidity**

**
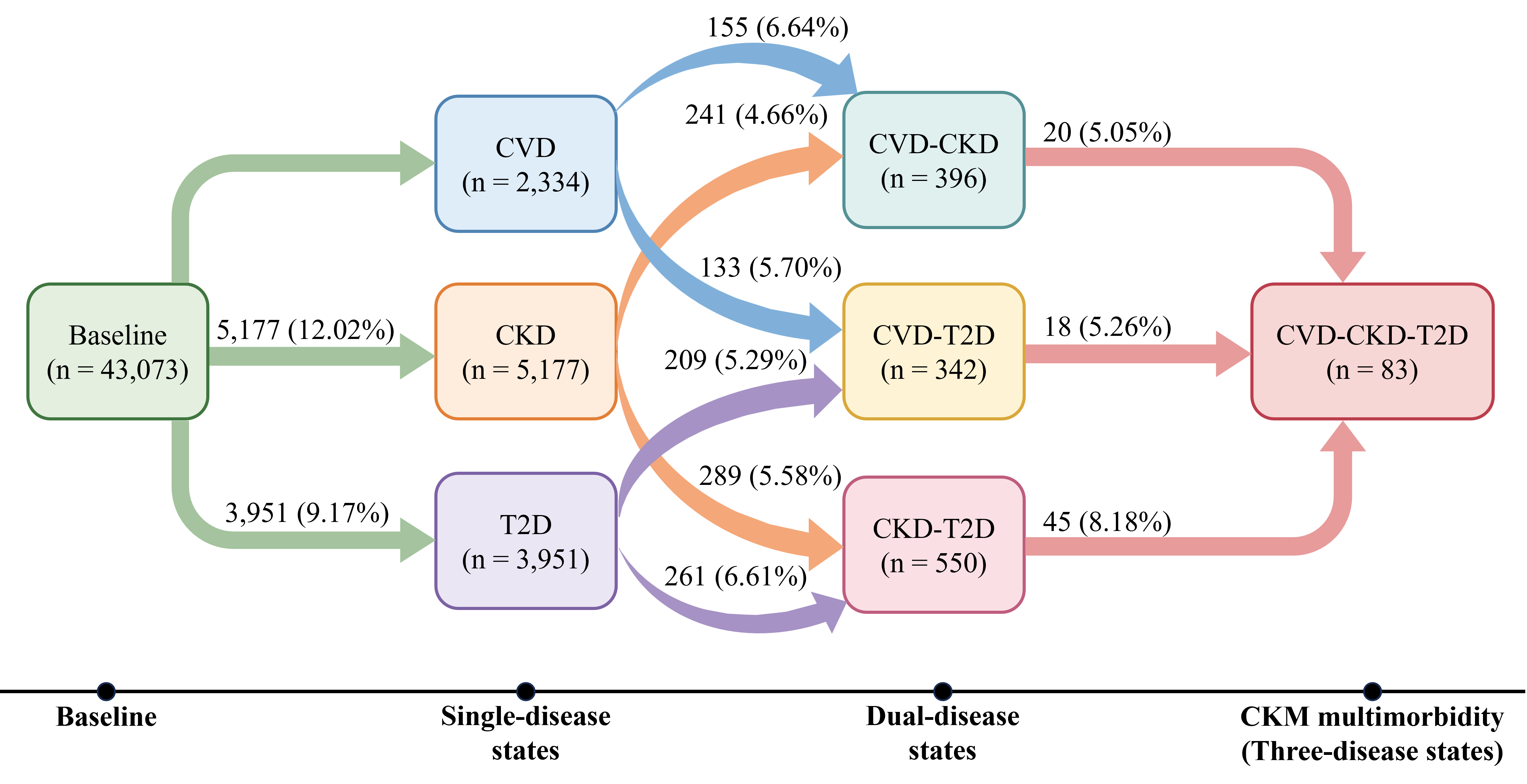
**

Abbreviations: CVD: cardiovascular disease; T2D: type 2 diabetes; CKD: chronic kidney disease.

**Supplemental Figure 4. Visualization of Transition Patterns of IFG Progression under Different Classification Criteria**


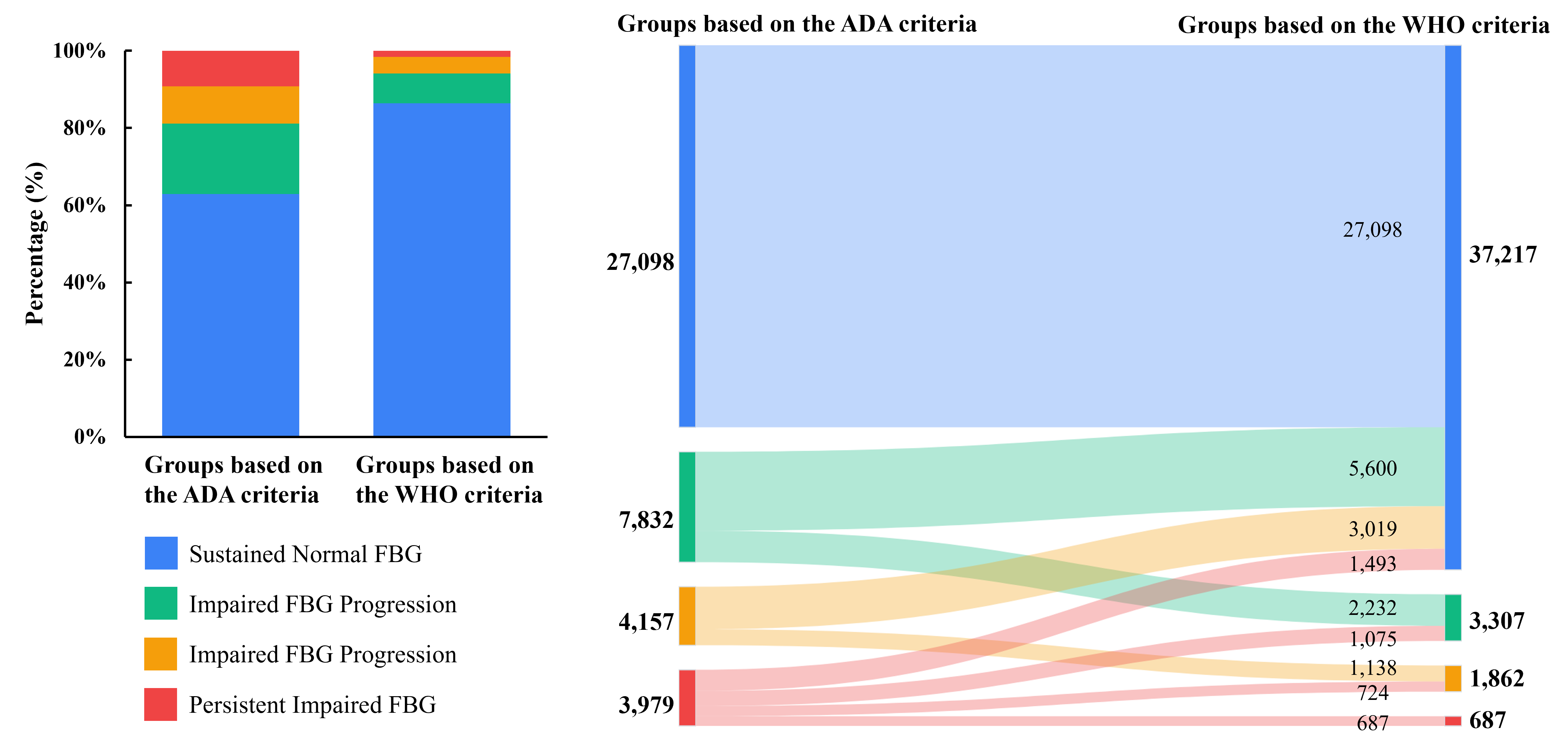


Abbreviations: IFG: impaired fasting blood glucose; FBG: fasting blood glucose; ADA: American Diabetes Association; WHO: World Health Organization.
